# Carbon dot superoxide dismutase nanozyme enhances reactive oxygen species scavenging in diabetic skin wound repair

**DOI:** 10.1016/j.jare.2025.03.049

**Published:** 2025-03-26

**Authors:** Zhu Yan, Yufei Zhang, Qin Chen, Jing Li, Xiaoying Ning, Fan Bai, Yaqi Wang, Xiaoming Liu, Yale Liu, Mingzhen Zhang, Cui Liu, Yumin Xia

**Affiliations:** a Department of Dermatology, The Second Affiliated Hospital of Xi’an Jiaotong University, Xi’an 710004, China; b Chongqing Key Laboratory of Natural Product Synthesis and Drug Research, Innovative Drug Research Center, School of Pharmaceutical Sciences, Chongqing University, Chongqing 400044, China; c Department of Dermatology, The Second Affiliated Hospital of Zhejiang University School of Medicine, Hangzhou 310009, China; d Department of Dermatology, Southern University of Science and Technology Hospital, Shenzhen 518055, China; e School of Basic Medical Sciences, Xi’an Jiaotong University, Xi’an 710061, China

**Keywords:** Diabetic wound, Carbon dot nanozyme, Antioxidation, Reactive oxygen species, Animal model

## Abstract

**Introduction:**

The accumulation of reactive oxygen species (ROS) in diabetic wounds leads to inflammation and impaired neovascularization. Recent studies have indicated that carbon dot nanozymes (C-dots) exhibiting superoxide dismutase (SOD)-like activity can neutralize excessive ROS and mitigate diseases associated with oxidative stress.

**Objectives:**

Our study was designed to evaluate the therapeutic impact of C-dots on the healing of diabetic wounds and to unravel the complex molecular mechanisms through which these nanozymes modulate oxidative stress and inflammatory responses within the wound microenvironment.

**Methods and results:**

We synthesized C-dots from carbon fiber and confirmed their structure using transmission electron microscopy. The presence of carbon–carbon double bonds on the C-dots was verified with X-ray photoelectron spectroscopy. We assessed the scavenging capacity of C-dots for superoxide anion, hydroxyl radical, and nitric oxide radical using electron spin resonance spectroscopy. Their SOD-like activity and total antioxidant capacity were evaluated with commercial assay kits. *In vitro* experiments showed that C-dots effectively scavenged excessive ROS, protecting human keratinocytes, vascular endothelial cells, and fibroblasts from oxidative stress-induced damage. Concurrently, C-dots increased the migratory capacity of fibroblasts. In a streptozocin-induced diabetic mice model, C-dots application enhanced skin wound healing, evidenced by accelerated re-epithelialization and orderly collagen matrix assembly. Mechanistic investigations indicated that C-dots markedly suppressed ROS generation and diminished the levels of inflammatory cytokines in the wound environment. Additionally, C-dots induced an M2 polarization phenotype in macrophages and promoted neovascularization, indicating a transition from the inflammatory to the proliferative phase. Quantitative proteomic analysis was conducted to further clarify the underlying mechanisms of C-dots in ameliorating diabetic wounds.

**Conclusion:**

C-dots represent a robust nanomaterial-based strategy for treating diabetic wounds, with the ability to accelerate healing by alleviating oxidative stress, mitigating harmful inflammatory responses, and fostering angiogenesis. This highlights their significant therapeutic potential in the field of biomedicine.

## Highlights

• The engineered C-dots exhibit superior stability, facile synthesis, and high scalability.
• C-dots protect skin-resident cells against oxidative stress-induced injury.
• Topical application of C-dots significantly accelerates the healing of diabetic wounds in mice.
• C-dots potently scavenge ROS and facilitate the M2 polarization of macrophages within the wound milieu.

## Graphical abstract

**Schematic illustration of the synthesis and application of C-dots for diabetic wound healing. a** The synthetic route of C-dots. **b** The schematic diagram illustrating the acceleration of diabetic wound healing through the treatment with C-dots.

**Figure:**
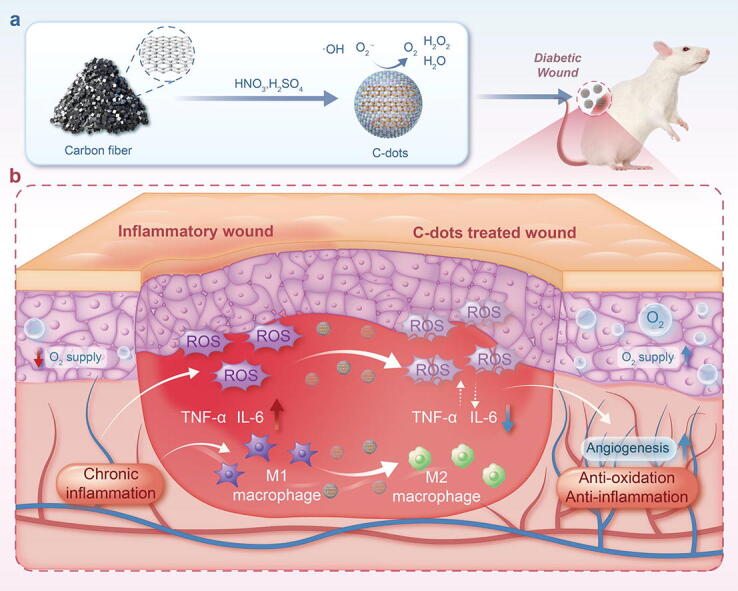

## Introduction

Epidemiological studies have shown that approximately 537 million people worldwide are afflicted with diabetes, with its prevalence expected to increase by 46% by the year 2045 [1], [2]. This rise is directly linked to a surge in major complications such as chronic wounds and gangrene, which are prevalent among diabetic individuals [3]. Specifically, about 25% of people with diabetes are at constant risk of chronic inflammation and persistent wounds, leading to non-traumatic limb amputations and significantly higher mortality rates worldwide [4], [5]. Under existing therapeutic methods, the 5-year survival rate for diabetic foot ulcer patients is a concerning 50%, which increases to 80% after amputation [6], [7]. These alarming statistics underscore the urgent need for innovative strategies to enhance the healing process of diabetic wounds and improve patient outcomes.

Current treatments, such as glucose management, oxygen therapy, debridement, antibiotics, and tissue grafts, are effective at containing the spread of diabetic wounds and infection [8], [9]. However, these methods often prove insufficient in facilitating complete wound healing due to the elevated recurrence rates of diabetic wounds [10]. The predominant barrier to effective tissue repair stems from heightened oxidative stress coupled with exacerbated inflammatory responses [11], [12], [13]. In diabetes, high blood sugar leads to protein hyperglycosylation and increased advanced glycosylation end products, which elevate reactive oxygen species (ROS) in immune cells and attract more monocytes/macrophages [14]. This disrupts the transition of macrophages from pro-inflammatory M1 to anti-inflammatory M2 states, causing a chronic inflammatory response that impedes angiogenesis and wound healing [15]. Moreover, the uncontrolled accumulation of ROS profoundly impairs the proliferation and differentiation of endogenous stem cells, functional cells, and growth factors within the wounded tissue, thereby significantly diminishing their regenerative capacity [16], [17], [18]. In conclusion, oxidative stress disrupts physiological healing processes, thereby contributing to the development of treatment-resistant chronic wounds.

To address these challenges, researchers have explored the use of antioxidant enzymes to restore the disrupted redox balance [19], [20], [21]. However, natural enzymes are prone to denaturation under physiological conditions, have complex synthesis processes, and are costly to produce, limiting large-scale manufacturing [22], [23]. In contrast, nanozymes—nanomaterials with enzyme-mimicking activities—offer significant advantages [24]. They can simulate the catalytic functions of natural enzymes by relying on their surface chemical properties and redox capabilities, allowing them to interact with substrates and catalyze reactions through electron transfer [25], [26]. Unlike natural enzymes, nanozymes resist denaturation and maintain consistent performance across various environments [27]. They are easily synthesized and modified for tailored applications, and can be produced at a large scale more easily, thus overcoming the challenges of natural enzyme preparation and manufacturing [28], [29]. These attributes make nanozymes a more practical and affordable alternative for managing diabetic wounds.

Current research on nanozymes for diabetic wound healing still faces significant limitations. Some nanozymes may induce side effects with prolonged use, and certain studies have not adequately addressed the multiple pathological mechanisms involved in wound healing [30]. In response to the critical need for safe and effective treatments in diabetic wound healing, this study underscores the application of a novel carbon dot nanozyme (C-dot). Unlike many nanozymes, C-dots exhibit stable and potent superoxide dismutase (SOD)-like activity, maintaining their effectiveness across a range of temperatures and diverse pH environments [31]. They can effectively neutralize harmful ROS, converting them into harmless substances and simultaneously reducing inflammatory cytokines, thereby mitigating conditions associated with oxidative stress and inflammation, such as ischemic stroke and hepatic ischemia–reperfusion injury [31], [32]. However, the potential application of C-dots in diabetic wound management remains unexplored. Given their excellent biocompatibility, reliable antioxidant and anti-inflammatory properties, C-dots are promising as a highly effective approach for addressing the multifaceted challenges in diabetic wound care [33].

This investigation aims to fill this research gap by exploring the therapeutic efficacy of C-dots in facilitating diabetic wound healing. By elucidating the mechanisms through which C-dots modulate oxidative stress and inflammatory responses within the wound environment, we hope to demonstrate their unique value in improving diabetic wound outcomes and highlight their potential as a transformative therapeutic strategy in this challenging clinical area.

## Materials and methods

### Synthesis of C-dots

The boiling solution, consisting of 50 mL of mixed acid with a 2:1 ratio of V(HNO_3_) to V(H_2_SO_4_), received an addition of 0.4 g of carbon fiber powder (Tansu Manufactory, Shanghai, China). For 3 h, the solution was kept boiling and the boiling temperature of the mixed acid solution was approximately 120℃. After boiling, the solution was cooled to room temperature over approximately 1 h. The resulting C-dots solution was then neutralized with NaHCO_3_ (Tianli, Tianjin, China). The specific duration for the neutralization step was about half an hour, and the quantity of NaHCO_3_ required for neutralization was about 100 g. The resultant neutralized mixture underwent purification via filtration through a 0.22 µm pore-sized membrane and subsequent dialysis spanning approximately seven days. Following dialysis, the C-dots solution was subjected to a secondary filtration utilizing a 0.22 μm hydrophilic membrane to remove any lingering particulate matter. Subsequently, the C-dots solution was concentrated and ultrafiltered employing a Millipore centrifugal filter unit equipped with a 100 kDa molecular weight cutoff membrane. Post-ultrafiltration, the fraction with a molecular weight below 100 kDa was isolated. After freeze-drying, a solid powder of C-dots was obtained, which can be stored at room temperature for a long period, ready for subsequent application.

### The SOD-like activity of C-dots

To evaluate the SOD-like activity of C-dots, we used a commercial WST-1 SOD Assay Kit (Dojindo, Kumamoto, Japan). WST-1 is a water-soluble tetrazolium salt that reacts with superoxide anions to generate a formazan dye exhibiting an absorbance peak at 450 nm. This assay is based on the principle that SOD or its mimetics inhibit the reduction of WST-1 by superoxide anions. The inhibition rate (%) is calculated according to the equation: $\frac{{(A}_{Control1}-A_{Control3})-{(A}_{\mathrm{Sample}}-A_{Control2})}{{(A}_{Control1}-A_{Control3})}\times 100\%$. The reaction was carried out at 37 °C. To generate an inhibition curve, the sample was subjected to serial dilution, and the dilution factor associated with 50% inhibition (IC_50_) was identified. Subsequently, the SOD-like activity of the sample was calculated by multiplying the IC_50_ dilution factor with the initial dilution factor of the sample. One unit (1 U) of SOD activity is specified as the quantity of enzyme in 20 μL of sample needed to achieve 50% inhibition of the WST-1 reduction reaction involving superoxide anions. The inhibition rate (%) and enzyme activity (U/mg) were calculated in accordance with the manufacturer’s guidelines.

### DPPH scavenging activity of C-dots

A Total Antioxidant Capacity Assay Kit with DPPH method (Macklin, Shanghai, China) was used to evaluate the antioxidant capacity of substances. We dissolved DPPH in methanol to create a solution with a known concentration of 0.15 mmol/L and prepared samples of C-dots with various concentrations. 100 μL samples of C-dots were added to each well containing an equal volume of DPPH solution. Then we allowed the reaction to proceed by incubating the mixture for 30 min in the absence of light. After incubation, the absorbance of the reaction mixture at 517 nm was measured using a spectrophotometer and the scavenging activity was calculated.

### ABTS scavenging activity of C-dots

The total antioxidant capacity of the C-dots was also assessed using a commercial Total Antioxidant Capacity Assay Kit with ABTS method (Beyotime, Shanghai, China). We mixed the ABTS and the oxidant solution in equal volumes to prepare a fresh working solution. This solution was then left to stand at room temperature in the dark for 12 to 16 h to ensure complete radical formation. Following this, we diluted the working solution as per the kit’s guidelines and added it to the samples of C-dots with various concentrations, and mixed thoroughly. After incubating for a specified time, the absorbance of the reaction mixture was measured at a wavelength of 734 nm to determine the extent of the antioxidant activity.

### Cell culture

HaCaT cells and HUVECs were procured from the China Center for Type Culture Collection. Both cell types were cultured in their respective media: HaCaT in DMEM with 10% FBS (Gibco, Grand Island, NY, USA), and HUVECs in ECM (ScienCell, Carlsbad, CA, USA). Primary mouse dermal fibroblasts were isolated from neonatal C57BL/6J mice and cultured in DMEM with 10% FBS. All cultures were maintained at 37 °C in a 5% CO_2_ humidified atmosphere, and the media were refreshed every 2–3 days.

### Intracellular ROS scavenging of C-dots

HaCaT cells, fibroblasts, and HUVECs were placed in 6-well plates and allowed to adhere for 12 h. Then they were exposed to varying concentrations of C-dots (0, 10, and 20 µg/mL) dispersed in complete media for a duration of 8 h. After the pretreatment, the culture media were refreshed, and the cells were treated with 1 mM H_2_O_2_ (Daxiong, Tianjin, China) for an additional hour to stimulate ROS generation. Subsequently, intracellular levels of ROS were determined using 2′,7′-dichlorofluorescein diacetate (DCFH-DA) (Beyotime, Shanghai, China) in accordance with the manufacturer’s instruction.

### Measurement of mitochondrial ROS

The mitochondrial ROS (mtROS) level was assessed using the MitoSOX Green fluorescent probe (Invitrogen, Carlsbad, CA, USA). Specifically, cells were exposed to varying concentrations of C-dots (0, 10, and 20 µg/mL) for a duration of 8 h and subsequently treated with 1 mM H_2_O_2_ for 1 h to induce mtROS production. After washing with PBS, the cells were stained with MitoSOX Green and analyzed using a confocal microscope following the manufacturer’s protocol.

### Cytoprotective effects of C-dots

HaCaT cells, fibroblasts, and HUVECs were placed in 96-well plates to adhere overnight. They were then exposed to C-dots at concentrations of 0, 10, and 20 µg/mL for 8 h. Afterward, cells were exposed to 1 mM H_2_O_2_ for 4 h to induce oxidative stress. The Cell Counting Kit-8 (CCK-8) (Beyotime, Shanghai, China) was then used to determine the cell viability, thereby assessing the protective impact of C-dots on cells.

### Fibroblast migration assessment

To examine the impact of C-dots on fibroblast migration, a wound-healing assay was conducted. Primary mouse dermal fibroblasts were grown in 12-well plates to the optimal density. Following the incubation period of 24 h, the culture medium was carefully removed and a consistent scratch was made across the surface of the well. Then fibroblasts were treated with C-dots dispersed in complete media at a concentration of 20 µg/mL for a specified duration. Cell migration into the wounded area was observed using an inverted microscope, and photographs were captured to document the healing process over time.

### Preparation of C-dots@Lotion

To make the solid C-dots suitable for application on the skin, we used a medical moisturizing dressing as the base lotion (provided by Xi’an Bohe Medical Technology Co., Ltd.) to dissolve the C-dots. This lotion, specifically designed for medical use, contains only purified water and sodium hyaluronate. C-dots were dissolved in the lotion by vigorous vortexing for 30 min to ensure uniform dispersion. The mixture was then continuously stirred for 2 h at room temperature to maintain homogeneity. After thorough homogenization, the C-dots were evenly dispersed in the lotion without any visible precipitation or stratification, resulting in a solution. To ensure the homogeneity of the mixture, the solution was vortexed periodically during storage to prevent sedimentation. Before each application to the wound site, the solution was vortexed again to ensure uniformity.

### *In vivo* biocompatibility analysis

All procedures involving animals were approved by Xi’an Jiaotong University Health Science Center Biomedical Ethics Committee (the animal experiment license number of the institution: 2024–2526). C57BL/6J mice (male, 8 weeks old) were individually raised in cages under standardized temperature conditions. To analyze the biocompatibility of C-dots, a 10-mm-diameter full-thickness skin wound was inflicted on the dorsal region. The mice were randomly assigned to three groups for the experiment, with one group serving as the control and receiving a 0.9% NaCl solution, one lotion-only group, and one group treated with C-dots@Lotion of 20 µg/mL. For 30 consecutive days, each mouse was administered 200 µL of its designated treatment directly to the wound site daily.

Following this treatment period, the mice were humanely euthanized, tissue sections from the heart, liver, spleen, lungs, and kidneys of the C-dots@Lotion, lotion, and control groups were prepared and stained with hematoxylin and eosin (H&E). The staining was employed to evaluate tissue morphology, and to scrutinize for indicators of adverse alterations, including inflammatory cell infiltration, hemorrhage/congestion, edema, fibrosis, necrosis, connective tissue proliferation, and calcification. Blood samples were collected from each group to perform a complete blood count including measurements of red blood cells, white blood cells, platelet counts, and hemoglobin concentrations. Serum biochemical assays included alanine aminotransferase (ALT), aspartate aminotransferase (AST), blood urea nitrogen (BUN), and creatine kinase (CK).

### Mouse model of diabetic wounds

C57BL/6J mice (male, 8 weeks old) received a daily intraperitoneal injection of STZ (Psaitong, Beijing, China) dissolved in sodium citrate buffer (0.1 mol/L, Solarbio, Beijing, China) at a dosage of 50 mg/kg for a period of five days. Tail vein blood was collected, and blood glucose levels were examined using a glucometer after 2 weeks. Mice exhibiting blood glucose concentrations above 16.7 mM were identified as diabetic and were kept under observation for a four-week period prior to the creation of skin wounds. The dorsal hairs of the diabetic mice were first shaved using a depilatory cream after anesthetization. Then, a circular full-thickness skin lesion, measuring 10 mm in diameter, was inflicted on the dorsal region of the mice. Subsequently, the diabetic mice with wounds were randomly assigned to three distinct groups: the control group (0.9% NaCl solution), the lotion group and C-dots@Lotion group of 20 µg/mL. Each group received their respective treatment daily, with a volume of 200 µL applied topically to the wound surface. Corresponding photographs were collected through the treatment period. The dimensions of the wound were determined utilizing ImageJ software (NIH, Bethesda, USA). The calculations were conducted as follows: Relative wound area (%) = Area_n_ / Area_0_ × 100%, where Area_0_ and Area_n_ are the wound areas on day 0 and on day 2, 5, 8, 10, or 12, respectively.

### Detection of ROS levels *in vivo*

To initially assess the *in vivo* ROS scavenging capability of C-dots, we utilized a dihydroethidium (DHE) probe (Beyotime, Shanghai, China) to measure ROS levels in the wound region on the 12th day. In the diabetic wound model, tissue samples were excised, prepared into cryosections, and subsequently stained with DHE. Fluorescence microscopy was employed to capture images, which were then analyzed using ImageJ software for quantification of ROS presence.

### H&E and Masson’s staining

Wound tissues from mice were collected and preserved in 4% paraformaldehyde. After undergoing ethanol dehydration, paraffin embedding, and sectioning, the tissues were stained with H&E. Besides, to evaluate collagen deposition, wound tissue samples were analyzed using Masson’s staining and then digitally scanned for analysis.

### Immunofluorescence analysis

Immunofluorescence was conducted in wound sections. Antibodies specific for 8-hydroxy-2′-deoxyguanosine (8-OHdG) and inducible nitric oxide synthase (iNOS) (Santa Cruz, CA, USA) were utilized to characterize oxidative stress levels. Antibodies for F4/80, CD206, and CD86 (Servicebio, Wuhan, China) were utilized to characterize macrophage phenotypes. To gauge inflammatory reactions, the wound sections were stained for tumor necrosis factor-alpha (TNF-α) and interleukin-6 (IL-6) with immunofluorescent antibodies (Abcam, Cambridge, UK). Angiogenesis within the wounds was evaluated by staining with antibodies against platelet endothelial cell adhesion molecule-1 (CD31) and α-smooth muscle actin (α-SMA) (Abcam, Cambridge, UK). Slides with staining were observed and visualized using a fluorescence microscope, and the intensity of fluorescence was measured with the aid of ImageJ software.

#### Western blotting

Proteins were extracted from the wounded tissue samples using RIPA buffer, and the protein concentration was determined using a BCA protein detection kit (Beyotime, Shanghai, China). Each well of the gel was loaded with 20 mg of protein, which were then subjected to electrophoresis and transferred onto a PVDF membrane (Millipore, Billerica, MA, USA). The membranes were probed and incubated with primary antibodies specific for iNOS (Abcam, Cambridge, UK), or beta-actin (Proteintech, Wuhan, China), followed by incubation with secondary antibodies. Band intensity was quantified using ImageJ software.

#### Enzyme-linked immunosorbent assay (ELISA)

Mouse skin tissue was washed with pre-cooled PBS to remove residual blood, weighed, and cut into small pieces using ophthalmic scissors. The tissue pieces were then ground in a glass homogenizer with pre-cooled PBS at a weight-to-volume ratio of 1:9 (e.g., 1 g of tissue to 9 mL of PBS). The homogenate was centrifuged at 5000 × g for 5–10 min at 4 °C. The resulting supernatant was used to detect the levels of 8-OHdG using an ELISA kit (Jianglai, Shanghai, China), following the manufacturer’s guidelines.

#### Statistical analysis

All data were shown as the mean ± standard error of mean (SEM) and analyzed with GraphPad Prism 9.0 (GraphPad Software, CA, USA). Student’s *t*-test was for two-group comparisons, and one-way analysis of variance (ANOVA) with Tukey’s test was for multiple groups. The number of experimental replicates was 3, and in the animal experiments, the number of samples per group was 5. Significance was set at *P* < 0.05, marked as *P < 0.05, **P < 0.01, ***P < 0.001, ****P < 0.0001.

## Results

### Preparation and characterization of C-dots

**Fig. 1:**
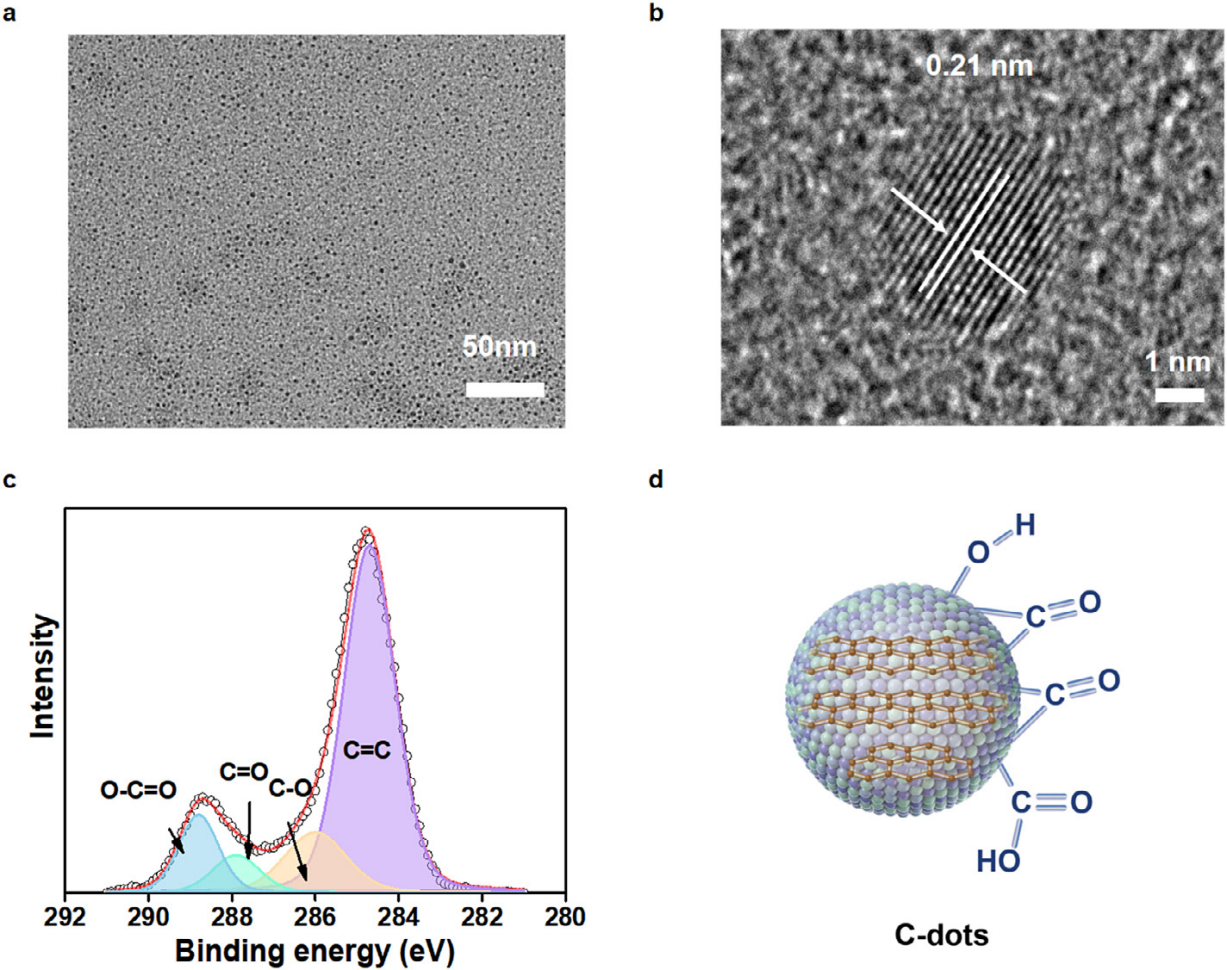
**Characterization of C-dots. a** The TEM image of C-dots. **b** The high-resolution TEM image and lattice of C-dots. **c** The XPS spectrum of C-dots. **d** The structure of C-dots with free functional groups.

Carbon fiber was used to create C-dots via an oxidation process utilizing a combination of nitric acid (HNO_3_) and sulfuric acid (H_2_SO_4_). With a carbon content exceeding 90%, carbon fiber embodies both the inherent characteristics of carbon materials and the pliable workability akin to textile fibers [34]. The currently recognized structure of carbon fiber is composed of two-dimensional, randomly oriented layers of graphite along the fiber axis [35]. The structure of the synthesized C-dots was examined through transmission electron microscopy (TEM). C-dots originating from carbon fiber displayed uniformity, measuring approximately 2.0±0.4 nm in diameter (Fig. 1**a**). High-resolution TEM images exposed distinct lattice fringes within the C-dots, with a mean interplanar distance of 0.21 nm, aligning with the documented (1 0 0) facet of graphite (Fig. 1**b**) [36]. X-ray photoelectron spectroscopy (XPS) results validated the presence of carbon–carbon double bonds (C=C), carbon–oxygen single bonds (C − O), carbonyl groups (C=O), and ester linkages (O − C=O) on their surface (Fig. 1**c and d**). The presence of C=C bonds implies a π-electron system, which might support electron transfer and stabilize intermediate species with unpaired electrons. Consequently, an adequate amount of C=C bonds is essential for enhancing the enzymatic activity.

**Fig. 2:**
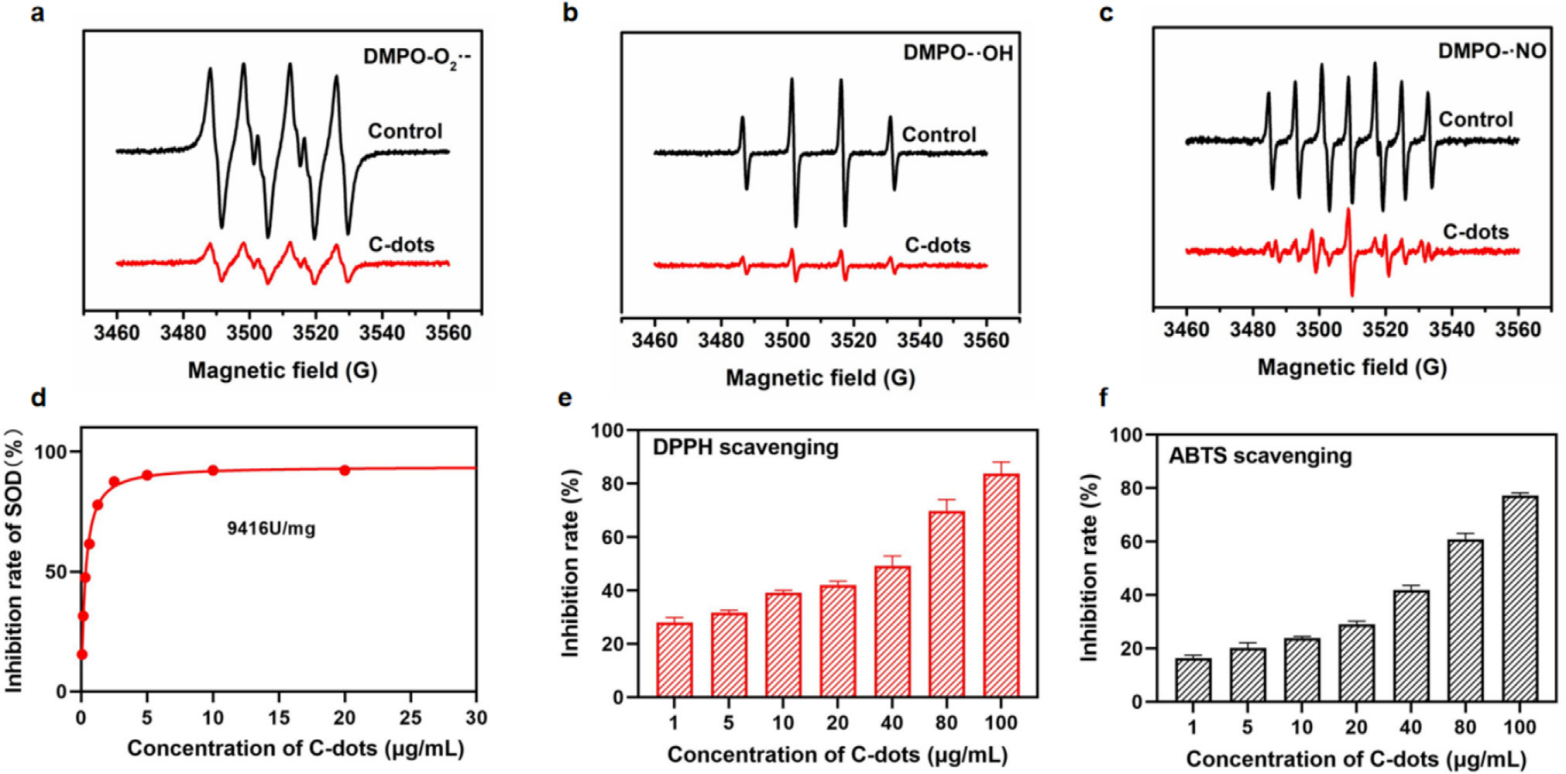
**Antioxidant activity of C-dots. a-c** ESR spectra of O_2_^•−^ (**a**), •OH (**b**), and •NO (**c**) scavenging by C-dots. **d** The SOD activity of C-dots. **e** DPPH radical scavenging activities of C-dots. **f** ABTS radical scavenging activities of C-dots. Data are presented as means ± SEM (n = 3).

Electron spin resonance (ESR) spectroscopy was employed to evaluate the O_2_^•−^, •OH and •NO scavenging ability of C-dots. The ESR signal significantly decreased in the presence of C-dots, indicating that C-dots could effectively eliminate O_2_^•−^ (Fig. 2**a**). Likewise, ESR results also indicated that C-dots were able to effectually scavenge •OH and •NO (Fig. 2**b and c**). Furthermore, the SOD-like activity of the C-dots was evaluated using a commercial SOD assay kit (WST-1). We tested the inhibition rates of C-dots at various concentrations (0.078125–40 µg/mL) and plotted the standard curve. As shown in Fig. 2**d, C**-dots achieved inhibition rates of 15% at 0.078125 µg/mL, 50% at 0.4425 µg/mL, and 90% at 5 µg/mL. These results indicate that the inhibition rate increased with concentration. Following the manufacturer’s guidelines, the SOD-like activity of C-dots was calculated to be as high as 9416 U/mg. Compared to a natural SOD enzyme that we tested, which exhibited an SOD activity of 6829 U/mg, C-dots clearly demonstrate a significantly higher SOD-like activity [37]. Surprisingly, the SOD-like activity of C-dots significantly surpasses that of previously reported SOD nanozymes, including Cu-SAzyme, MnPS_3_, and pero-nanozymes [38], [39], [40]. This variation might stem from the distinct structural characteristics of the carbon sources, density, porosity, and hardness.

DPPH stands for 1,1-Diphenyl-2-picrylhydrazyl Free Radical, which is used as a sensitive colorimetric free radical scavenger and serves as a general antioxidant detector. The DPPH assay is based on the principle that the purple-colored DPPH radical solution decolorizes upon interaction with an antioxidant, with the extent of decolorization proportional to the antioxidant capacity of the tested substance. As demonstrated in Fig. 2**e**, the inhibition rate of DPPH radicals increased with the rising concentration of C-dots. Specifically, the inhibition rate reached 50% at a concentration of 40 µg/mL and exceeded 80% at 100 µg/mL. This concentration-dependent increase in inhibition rate highlights the antioxidant potential of C-dots. Besides, a Total Antioxidant Capacity Assay Kit was used to thoroughly assess the antioxidant potential of the C-dots. As a colorant, 2,2′-azino-bis(3-ethylbenzthiazoline-6-sulfonic acid) (ABTS) can be oxidized to green ABTS + in the presence of an oxidizing agent but is inhibited in the presence of an antioxidant. The antioxidant capacity can therefore be derived by calculating the absorption of ABTS+ at 734 nm. In the ABTS assay, increased C-dots concentrations led to greater suppression of ABTS+ formation. C-dots could neutralize over 70% of the free radicals at a concentration of 100 μg/mL, confirming that they possess good ROS scavenging capability (Fig. 2**f**). Collectively, these findings clearly demonstrate that the C-dots possess notable antioxidant capabilities, with an enhanced capability to neutralize ROS as their concentration increases.

### *In vitro* biocompatibility of C-dots

Wound healing is a highly sophisticated process, in which the biological behavior of repair cells constitutes an essential part [41]. Upon injury, keratinocytes migrate to cover wounds, fibroblasts become myofibroblasts aiding contraction and tissue formation, and endothelial cells create new capillaries for blood supply to the healing area [42]. The new vascular networks supply a rich blood supply to connective tissue and epidermal cells. Consequently, we evaluated the cytotoxicity of C-dots on HaCaT cells, HUVECs, and fibroblasts using the CCK-8 assay. As depicted in **Supplementary Fig. 1a-c**, exposure to 20 μg/mL of C-dots for 24 h resulted in minimal effects on the viability of all three cell types, with cell survival rates exceeding 90%. Even after a 48-hour incubation with 100 μg/mL of C-dots, the viability of all three cell types remained above 80%. These findings indicate that C-dots possess excellent biocompatibility at the cellular level. Based on these results, 20 μg/mL was selected as the maximum concentration for all subsequent cell experiments.

Since the hemolytic toxicity of nanomaterials might interfere with their *in vivo* applications, we evaluated the hemocompatibility of C-dots using a hemolysis test. Erythrocytes were incubated with different concentrations of C-dots for 4 h, followed by centrifugation, and hemolysis was assessed by measuring the absorbance at 540 nm of the supernatant [43], [44]. As shown in **Supplementary Fig. 2**, there was no erythrocyte hemolysis following incubation with C-dots from 0 to 200 μg/mL. When erythrocytes were incubated with C-dots at concentrations ranging from 500 to 3000 μg/mL, the hemolysis rates remained below 5%, which is considered acceptable for biomaterials according to the American Society for Testing and Materials‌ (ASTM) standard (#F756-2017). Based on these findings, C-dots have demonstrated excellent hemocompatibility.

### *In vitro* antioxidant effects and cell migration promotion of C-dots

**Fig. 3:**
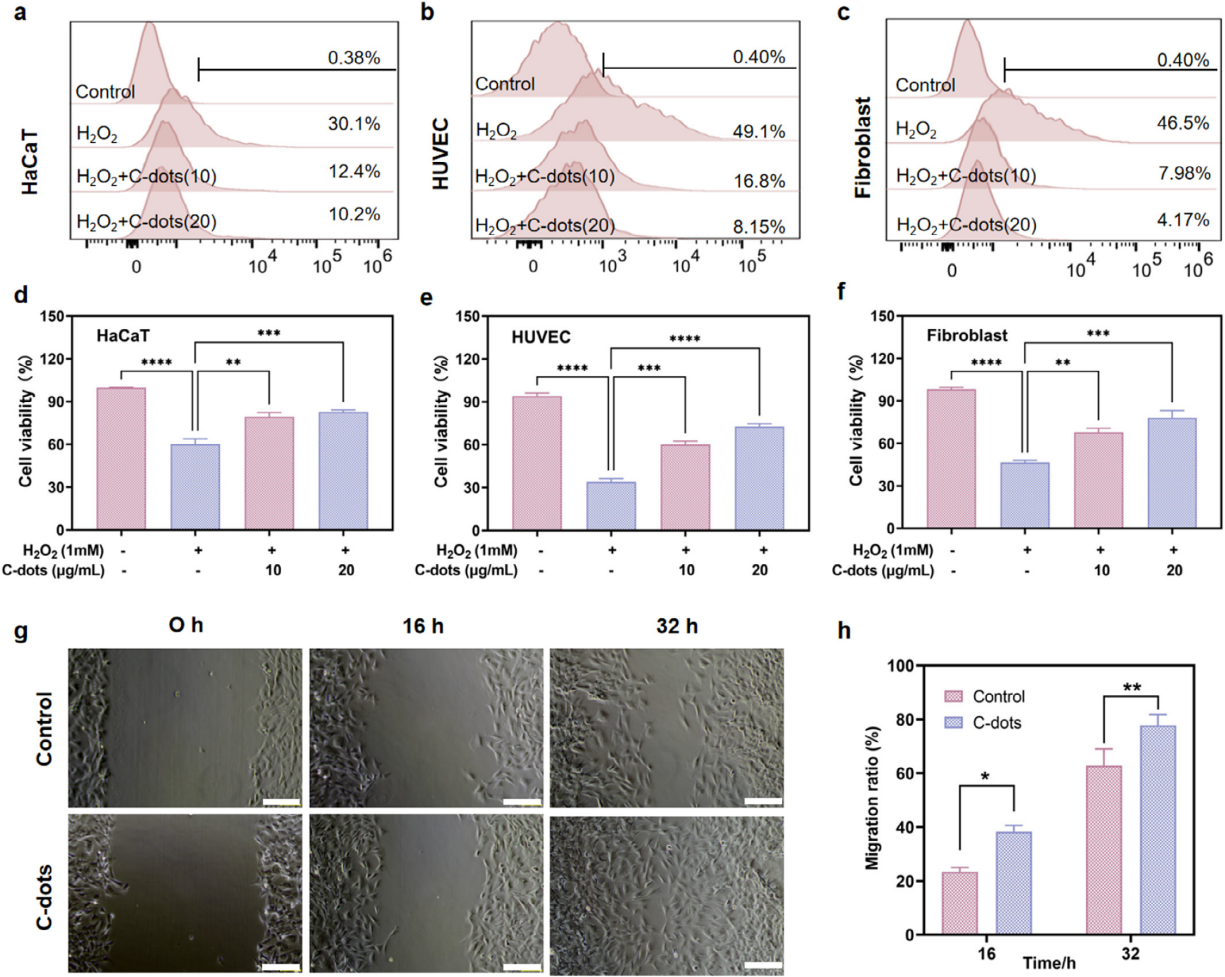
***In vitro* antioxidant effects and cell migration promotion of C-dots. a-c** Flow cytometry of HaCaT cells (**a**), HUVECs (**b**), and fibroblasts (**c**) stained with DCFH-DA after pretreatment with C-dots (0, 10, 20 µg/mL) for 8 h, followed by exposure to 1 mM H_2_O_2_ for 1 h. **d-f** Cell viability of HaCaT cells (**d**), HUVECs (**e**), and fibroblasts (**f**) after pretreatment with C-dots (0, 10, 20 µg/mL) for 8 h, followed by exposure to 1 mM H_2_O_2_ for 4 h. **g** Representative images of the wound gap over time in fibroblasts treated with and without C-dots at a concentration of 20 μg/mL. Scale bar: 200 μm. **h** Migration ratio (%) of fibroblasts at 16 and 32 h in the control and C-dots groups. Data are presented as means ± SEM (n = 3; * *P* < 0.05, ** *P* < 0.01, *** *P* < 0.001, **** *P* < 0.0001).

HaCaT cells, HUVECs, and fibroblasts were preincubated with different concentrations of C-dots (0, 10, 20 µg/mL) and then exposed to 1 mM H_2_O_2_ for 1 h to stimulate ROS generation. Then, the levels of ROS were assessed using a ROS Assay Kit with detection performed via flow cytometry. As a non-fluorescent permeable compound, DCFH-DA can be hydrolyzed by intracellular esterases to form 2′,7′-dichlorodihydrofluorescein, which can then be oxidized by intracellular ROS to generate fluorescent 2′,7′-dichlorofluorescein. Flow cytometry analysis demonstrated that HaCaT cells subjected directly to 1 mM H_2_O_2_ without prior C-dots incubation exhibited a pronounced increase in fluorescence intensity, corroborating the successful induction of elevated intracellular ROS levels. However, this increase was significantly mitigated by pre-incubation with 10 or 20 µg/mL C-dots (Fig. 3**a**). Comparable effects were observed in HUVECs and fibroblasts (Fig. 3**b and c**), indicating that C-dots are capable of efficiently eliminating ROS inside cellular environments.

We also investigated the potential of C-dots to shield cells against detrimental oxidative stress and enhance their viability under such conditions. HaCaT cells, HUVECs, and fibroblasts were preincubated with different concentrations of C-dots and then exposed to 1 mM H_2_O_2_ for 4 h to induce oxidative stress. Subsequently, cell viability was assessed using the CCK-8 assay. According to Fig. 3**d-f**, HaCaT cells, HUVECs, and fibroblasts treated with H_2_O_2_ for 4 h showed cellular mortality, with cell survival rates dropping to 60.13%, 33.89% and 46.55%, respectively. However, pre-treatment with 10 and 20 µg/mL of C-dots significantly improved the viability of all cell types, indicating their potential to alleviate the cytotoxic effects of oxidative stress.

To further elucidate the antioxidant impact of C-dots, we conducted an experiment using MitoSOX, a specific fluorescent probe for detecting mtROS. HaCaT cells, HUVECs, and fibroblasts were pre-incubated with different concentrations of C-dots and then exposed to 1 mM H_2_O_2_ for 1 h. After staining with MitoSOX Green and washing to remove unbound dye, the cells were analyzed using confocal laser scanning microscopy to visualize the levels of mtROS. The results revealed a significant increase in green fluorescence intensity in fibroblasts treated with 1 mM H_2_O_2_ alone, indicating elevated mtROS levels induced by H_2_O_2_. In contrast, preincubation with 10 or 20 µg/mL C-dots resulted in a marked reduction in mtROS levels **(Supplementary Fig. 3)**. Similarly, analogous effects were also observed in HaCaT and HUVEC cells (data not shown), which suggest that C-dots could also scavenge excessive mitochondrial superoxide radicals as well.

Moreover, the fibroblast migration assay was evaluated in the control group and the C-dots-treated group (20 μg/mL) at various time points (0 h, 16 h, and 32 h). Microscopic images revealed that the migration speed of fibroblasts into the scratched areas was enhanced when co-cultured with C-dots. Quantitatively, the migration ratio of C-dots-treated cells was approximately 1.4-fold higher than that of the control group at 16 h (*P* = 0.0199) and 1.3-fold higher at 32 h (*P* = 0.0081). These findings suggest that C-dots not only significantly enhance cell survival under oxidative stress but also moderately accelerate fibroblast migration, which is essential for tissue repair and regeneration.

### Biocompatibility of C-dots *in vivo*

Considering the critical importance of nanomaterial compatibility with biological systems for medical use, a comprehensive assessment of the biocompatibility of C-dots was carried out. To ensure the suitability of C-dots for skin application, a formulation was developed by dissolving the C-dots in a medical-grade moisturizing dressing consisting solely of purified water and sodium hyaluronate. Visual representations of C-dots in aqueous suspension at 20 µg/mL (C-dots@H_2_O), the lotion itself, and C-dots within the lotion at an equivalent concentration (C-dots@Lotion) are presented in **Supplementary Fig. 4**.

**Fig. 4:**
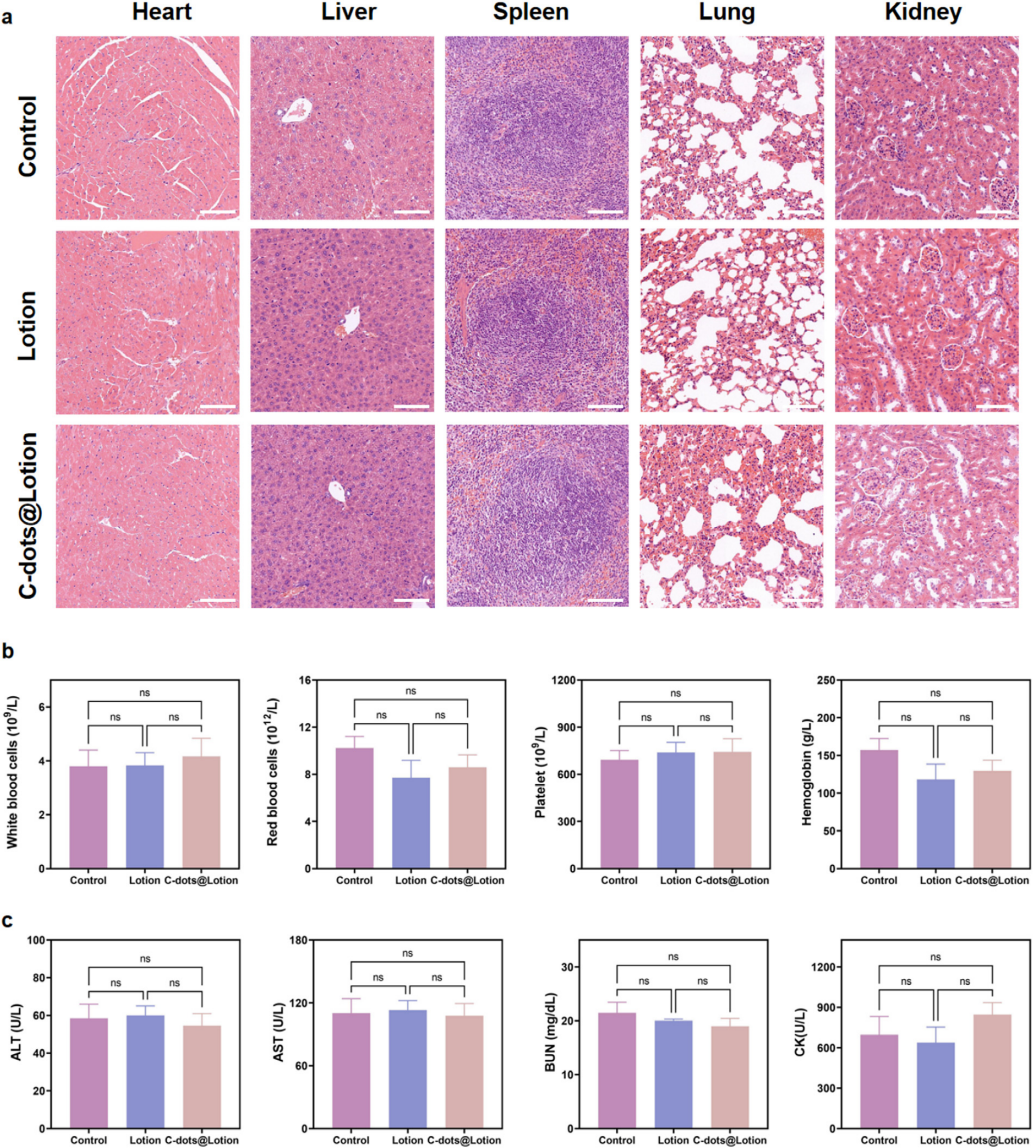
**Assessment of *in vivo* biocompatibility of C-dots. a** H&E staining of tissue sections from major organs of mice across different groups. Scale bar: 100 μm. **b** Routine blood index measurements for mice in the various groups. **c** Blood biochemical analysis results for mice in the various groups. Data are presented as means ± SEM (n = 3).

Then a full-thickness excisional wound, 10 mm in diameter, was created on the dorsal region of C57BL/6J mice, which were then randomly allocated into three groups: a control group treated with 0.9% NaCl solution, a lotion group, and a C-dots@Lotion group at 20 µg/mL. The mice received daily treatments for 30 days prior to euthanasia, after which major organs were collected for H&E staining. As depicted in Fig. 4**a**, histopathological examination revealed no significant differences in organ tissues between the C-dots@Lotion and control groups. Specifically, heart tissues showed normal myofiber arrangement and connective tissue without signs of inflammation or calcification. Liver tissues were free from steatosis, inflammation, fibrosis, or necrosis. Spleens exhibited uniform structure without hemorrhage or congestion. Lung tissues were similar across groups in terms of exudates and cellular infiltration. Kidney tissues in the C-dots@Lotion group were comparable to controls with respect to inflammation and tissue proliferation. These results indicate that the topical administration of C-dots@Lotion at a concentration of 20 μg/mL did not induce significant adverse toxicity in major organs over a 30-day period.

Additionally, blood parameters including red blood cells, white blood cells, platelet counts, and hemoglobin concentrations were comparable across the C-dots@Lotion, lotion, and control groups (Fig. 4**b**). Standard biochemical markers, such as alanine aminotransferase (ALT), blood urea nitrogen (BUN), creatine kinase (CK), and aspartate aminotransferase (AST), also showed no significant differences (Fig. 4**c**). Collectively, these findings support the favorable *in vivo* biocompatibility of C-dots at the therapeutic dose.

### C-dots accelerate healing of experimental diabetic wounds in mice

*In vitro* studies have demonstrated that C-dots could scavenge ROS, thereby shielding cells from damage and exhibiting favorable biocompatibility. Motivated by these findings, we aimed to investigate the therapeutic efficacy of C-dots in promoting diabetic wound healing using a murine model.

**Fig. 5:**
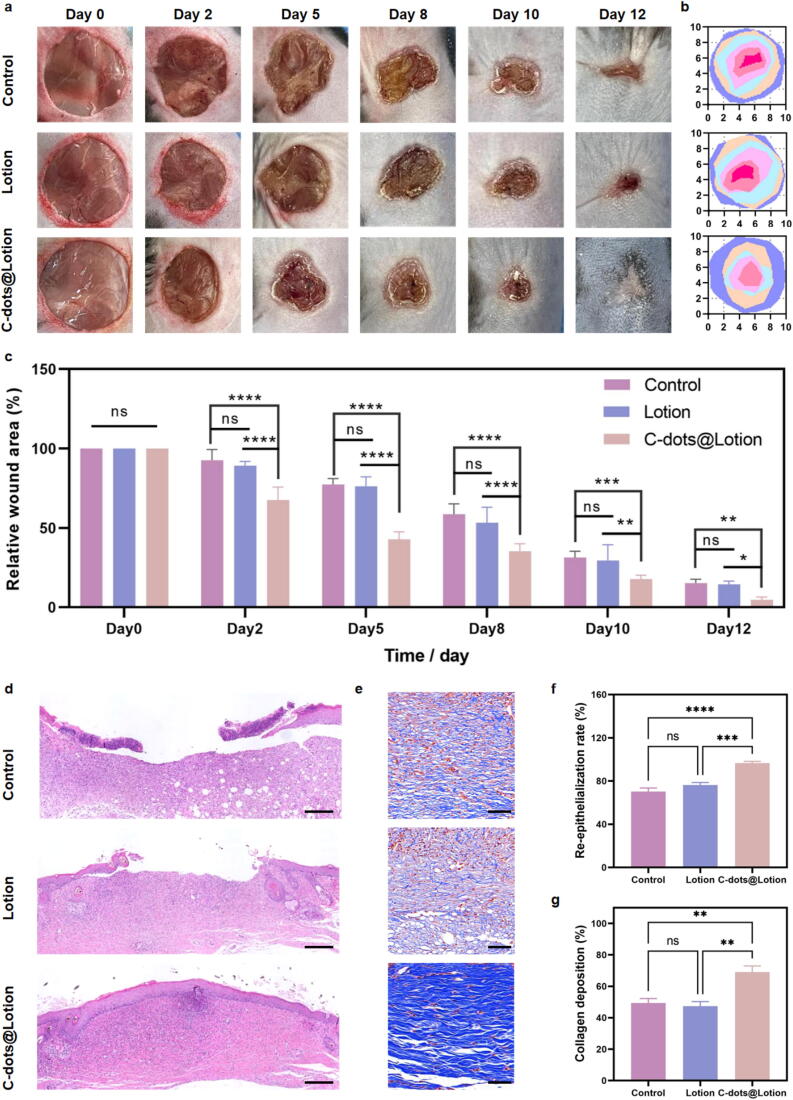
***In vivo* effect of C-dots on diabetic wound healing. a** Representative photographs of the wound healing process. **b** Schematic diagram of the wound trace. **c** Statistical analysis of relative wound area. **d** H&E staining of wound tissues. Scale bar: 200 µm. **e** Masson’s trichrome staining of wound tissues. Scale bar: 50 µm. **f** Statistical analysis of the re-epithelialization rate. **g** Statistical analysis of collagen deposition. Data are presented as means ± SEM (n = 5; * *P* < 0.05, ** *P* < 0.01, *** *P* < 0.001, **** *P* < 0.0001).

A type 1 diabetes murine model, characterized by fasting blood glucose levels consistently surpassing 16.7 mM, was induced using STZ following established protocols [45], [46]. Subsequently, a 10 mm diameter punch was used to inflict a full-thickness wound on the dorsal skin. The mice were allocated into distinct treatment groups: a control group treated with 0.9% NaCl solution, a lotion group, and a C-dots@Lotion group at a concentration of 20 µg/mL. The wounds were monitored at regular intervals, and photographic documentation was obtained throughout the treatment duration. All wounds demonstrated a reduction in size over time, with no observable signs of infection. Comparative wound site imagery revealed that the wounds in the C-dots@Lotion group were notably smaller compared to those in the other two groups, exhibiting near-complete closure and re-epithelialization by day 12, whereas wounds in the control and lotion groups remained open, accompanied by dark-red granulation tissue (Fig. 5**a and b**). To quantitatively assess the healing efficacy, the relative wound area was measured at each observation time point. Throughout the treatment period, the C-dots@Lotion group exhibited the most rapid healing rate, with a healing extent surpassing 50% by day 5, approximately 80% by day 10, and approaching full closure by day 12. In stark contrast, wounds in the control and lotion groups remained unhealed on day 12. Notably, no significant differences in wound closure rates were observed between the control and lotion groups during the healing process, indicating that the lotion alone did not exert a significant impact on diabetic wound healing (Fig. 5**c**).

Subsequently, on day 12 post-treatment, wound tissues were excised from the treated mice for histological examination to evaluate the efficacy of wound repair and tissue regeneration. Re-epithelialization and granulation tissue formation are pivotal for gauging successful wound healing. Histological evaluation via H&E staining revealed that the group treated with C-dots@Lotion exhibited a diminished wound area and an accelerated re-epithelialization rate when contrasted with the control and the group treated with lotion alone (Fig. 5**d and f**). During the phase of tissue remodeling, fibroblasts migrate to the wound site, where they deposit collagen, thereby facilitating the formation of the extracellular matrix. To quantitatively assess collagen deposition among the treatment groups, Masson’s trichrome staining was employed. The collagen fibers in the C-dots@Lotion group were markedly more extensive, dense, and exhibited superior alignment compared to those in the other groups. Conversely, there was no noteworthy variation in collagen deposition between the control and lotion groups (Fig. 5**e and g**).

The body weight of the mice was meticulously monitored throughout the healing period. Observations showed no significant changes in body weight at days 0, 2, 5, 8, 10, and 12 post-treatment across all three groups (**Supplementary Fig. 5**). This finding indicated that the administration of C-dots@Lotion exerted no direct influence on the body weight of the mice. In conclusion, the heightened rate of re-epithelialization and intensified collagen fiber synthesis within the C-dots@Lotion group underscore the significant influence of C-dots on the acceleration of the wound healing cascade.

### C-dots mitigate oxidative stress and foster angiogenesis in mice

**Fig. 6:**
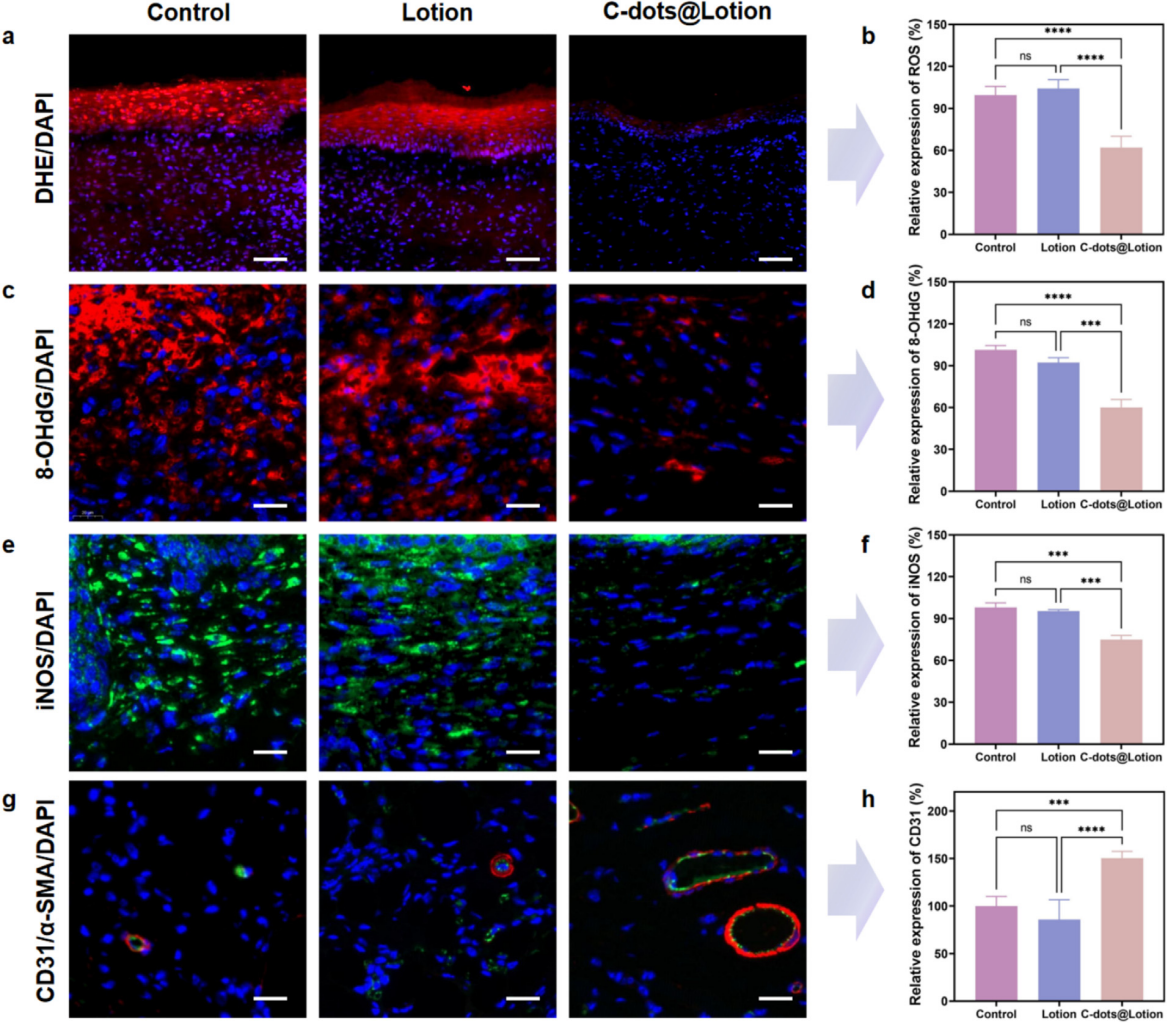
**The impact of C-dots on oxidative stress and angiogenesis. a, b** DHE staining images of different treatment groups (**a**) and quantitative analysis of wound ROS levels measured as a relative percentage compared to the control group (**b**). **c, d** Immunofluorescence detection of 8-OHdG (**c**) and quantitative analysis of 8-OHdG expression (**d**). **e, f** Immunofluorescence detection of iNOS (**e**) and quantitative analysis of iNOS expression (**f**). **g, h** Immunofluorescent labeling was performed for CD31 (in red) and α-SMA (in green) (**g**) and quantitative analysis of CD31 expression (**h**). Scale bar: 25 µm. Data are presented as means ± SEM (n = 5; * *P* < 0.05, ** *P* < 0.01, *** *P* < 0.001, **** *P* < 0.0001).

To elucidate the capacity of C-dots in mitigating oxidative stress, a series of *in vivo* experiments were conducted, demonstrating the efficacy of C-dots in scavenging ROS and reducing oxidative damage. Initially, the ROS scavenging efficacy of C-dots was evaluated using a DHE probe to quantify ROS levels in the wound area on day 12 post-surgery. Fluorescence imaging was employed to visualize ROS presence, with the mean fluorescence intensity (MFI) subsequently measured. The control group exhibited intense red fluorescence, indicative of high ROS levels, while the C-dots@Lotion group showed a significant reduction in signal intensity, a reduction that was not observed in the lotion-only group (Fig. 6**a and b**). These findings suggest that C-dots effectively scavenge ROS in the wound microenvironment.

To further assess the impact of C-dots on oxidative stress, the expression of 8-OHdG and iNOS was examined. 8-OHdG, an oxidative DNA adduct formed when ROS attack guanine bases, serves as a biomarker for oxidative DNA damage [47], [48]. Fluorescence imaging revealed strong red fluorescence in both the control and lotion groups, indicating high levels of oxidative DNA damage. In contrast, the C-dots@Lotion group exhibited significantly reduced fluorescence intensity, suggesting lower 8-OHdG expression (Fig. 6**c and d**). This finding was supported by ELISA results, which measured 8-OHdG levels in wound tissues across all groups. The C-dots@Lotion group exhibited significantly reduced 8-OHdG levels compared to both the control and lotion groups, offering quantitative evidence of diminished oxidative stress (**Supplementary Fig. 6**).

iNOS, typically quiescent under normal conditions, is upregulated by inflammatory stimuli and leads to the production of nitric oxide, serving as an indicator of oxidative and nitrosative stress. It is also a hallmark of the proinflammatory M1 macrophage phenotype [49]. Immunofluorescence staining for iNOS revealed strong green fluorescence in both the control and lotion groups, while the C-dots@Lotion treatment demonstrated attenuated fluorescence intensity, indicative of reduced iNOS expression **(**Fig. 6**e and f)**. Western blot analysis further validated that the expression of iNOS in the wound tissues of mice treated with C-dots@Lotion was significantly lower compared to both the control and the lotion-only groups (**Supplementary Fig. 7**). Collectively, these findings provide compelling evidence that C-dots effectively mitigate oxidative stress by scavenging ROS and modulating the expression of oxidative stress biomarkers.

To assess angiogenesis within wound sections, immunofluorescence staining was conducted using CD31 (red fluorescence), a marker specific for endothelial cells, and α-SMA (green fluorescence), a marker for smooth muscle cells. CD31-positive and α-SMA-positive staining indicates the presence of mature blood vessels, whereas CD31-positive and α-SMA-negative staining denotes the presence of immature blood vessels [50]. Typically, the 12-day mark corresponds to the wound remodeling stage, a period characterized by an abundance of mature blood vessels. The control and lotion groups exhibited diminished red fluorescent signals (CD31) and green fluorescent signals (α-SMA), indicating a scarcity of mature blood vessels. In stark contrast, the C-dots@Lotion group displayed significantly intensified fluorescence for both markers, a finding corroborated by quantitative analysis (Fig. 6**g and h**). The C-dots@Lotion group exhibited a higher number of mature blood vessels compared to the control and lotion groups. Moreover, the mature vessels in the C-dots@Lotion group were markedly larger and displayed tubular structures, indicative of enhanced angiogenesis (Fig. 6**g**). Taken together, these observations underscore the potent angiogenic effects of C-dots, suggesting that they can markedly promote wound healing by effectively mitigating oxidative stress and bolstering angiogenesis.

### C-dots modulate macrophage polarization and alleviate inflammation during wound repair

**Fig. 7:**
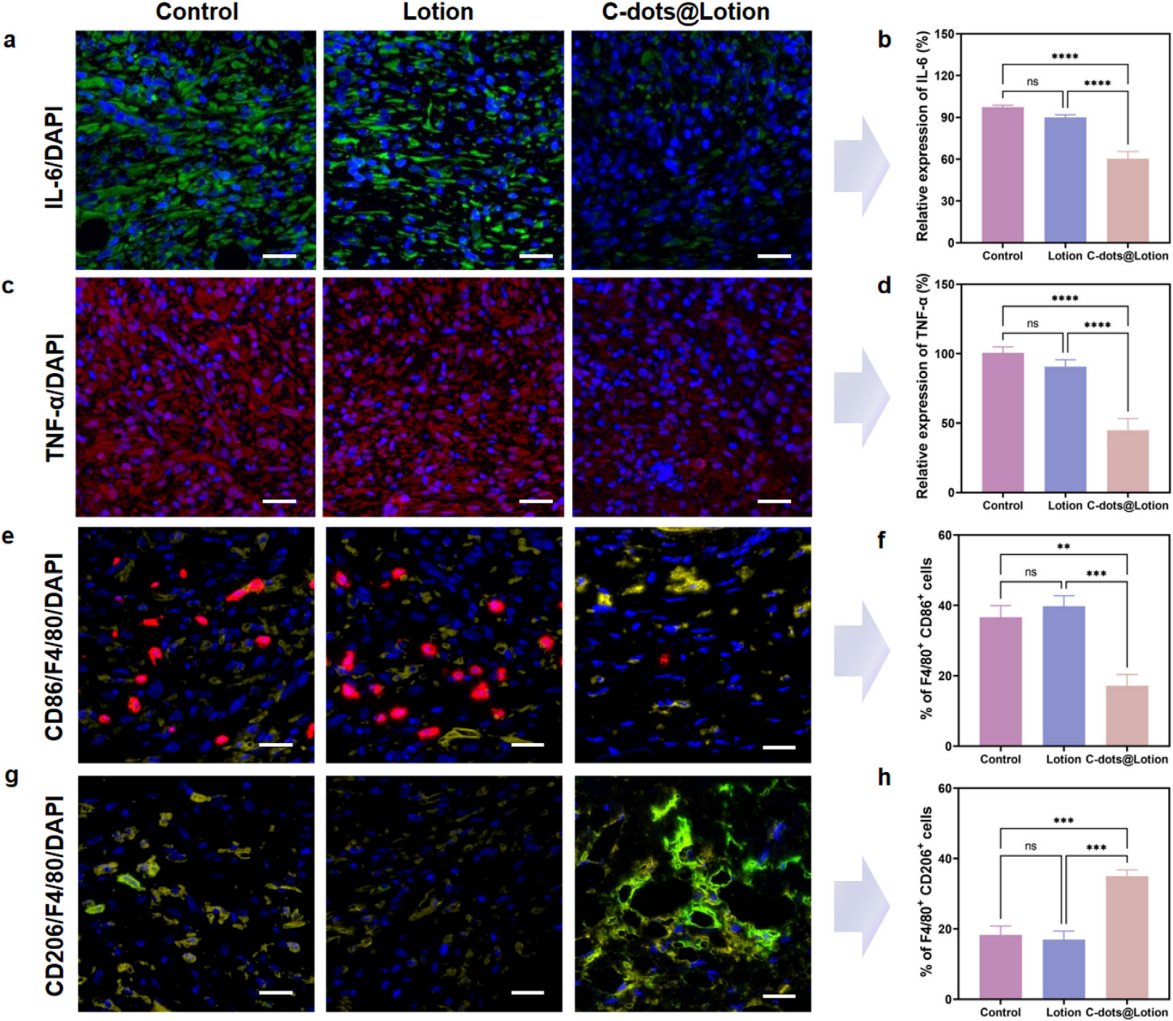
**The modulation of macrophage polarization and inflammation by C-dots. a, b** Immunofluorescence detection of IL-6 (**a**) and quantitative analysis measured as a relative percentage compared to the control group (**b**). **c, d** Immunofluorescence detection of TNF-α (**c**) and quantitative analysis measured as a relative percentage compared to the control group (**d**). **e, f** Immunofluorescent identification of M1 phenotype macrophages marked by CD86 (red) and F4/80 (yellow) (**e**), and statistical representation of M1 macrophage proportion (**f**). **g, h** Immunofluorescent identification of M2 phenotype macrophages marked by CD206 (green) and F4/80 (yellow) (**g**), and statistical representation of M2 macrophage proportion (**h**). Scale bar: 25 µm. Data are presented as means ± SEM (n = 5; * *P* < 0.05, ** *P* < 0.01, *** *P* < 0.001, **** *P* < 0.0001).

The hyperglycemic state characteristic of diabetes mellitus frequently precipitates a sustained inflammatory response in chronic diabetic wounds. To elucidate the influence that C-dots have on this inflammatory milieu, we examined the levels of two key inflammatory cytokines, TNF-α and IL-6, through immunofluorescence staining. Both the control and lotion groups exhibited intense fluorescence signals for both IL-6 and TNF-α, indicative of a pronounced inflammatory reaction (Fig. 7**a and c**). In contrast, the C-dots@Lotion group displayed attenuated expression levels of these cytokines, as illustrated by the statistical analysis of relative expression levels (Fig. 7**b and d**). These results indicate that C-dots are capable of reducing the inflammatory cytokines in diabetic wounds.

In diabetic wounds that fail to heal, there is a predominance of proinflammatory M1 macrophages, while the transition to the anti-inflammatory M2 phenotype is markedly hindered [51]. To evaluate the inflammatory dynamics within diabetic wounds following various treatments, we employed immunofluorescence staining with F4/80 (indicating macrophages, stained yellow), CD86 (indicating M1 macrophages, stained red), and CD206 (indicating M2 macrophages, stained green) to visualize the presence of M1 and M2 macrophages. The levels of CD86 and CD206 were quantified and analyzed to determine the effects of C-dots on macrophage polarization. We observed that in the control and lotion groups, wounds exhibited intense red fluorescence, indicative of substantial M1 macrophage infiltration and an inflammatory milieu. Conversely, the C-dots@Lotion group displayed diminished red fluorescence intensity, signifying a notable decrease in M1 macrophage presence (Fig. 7**e and f**). These findings align with previous observations of reduced levels of IL-6, TNF-α, and iNOS in the C-dots treated group, as they are closely associated with M1 macrophage-mediated inflammation.

M2 macrophages play a crucial role in tissue repair by balancing proinflammatory and anti-inflammatory functions, driving angiogenesis, and aiding in collagen deposition within the wound bed [52]. Post-treatment with C-dots@Lotion, a notable enhancement in the green fluorescence intensity of the M2 macrophage marker CD206 was observed, indicative of an increased M2 macrophage presence (Fig. 7**g and h**). In contrast, treatment with the lotion alone did not elicit significant changes in the expression levels of either CD86 or CD206, suggesting that the lotion lacks intrinsic anti-inflammatory properties. These observations imply that C-dots robustly influence the transition of macrophages from the M1 phenotype to the M2 phenotype, thereby potentially aiding in the shift of diabetic wounds from the inflammatory phase to the proliferative phase. These aforementioned findings offer a compelling rationale for the superior healing dynamics and reduced healing time observed in the C-dots@Lotion group within the *in vivo* wound healing experiments.

### Proteomic analysis of wound tissues with C-dots treatment

To further elucidate the mechanisms through which C-dots improve diabetic wounds, we performed a quantitative proteomic analysis on wound tissue samples from three distinct groups of mice: non-diabetic healthy controls (Control), STZ-induced diabetic mice (Diabetes), and C-dots@Lotion treated diabetic mice (C-dots) [53]. The aim was to identify changes in proteins and pathways involved in wound healing, leading to the identification of a total of 8055 proteins across the groups, with differential expression analysis revealing proteins with a fold change of |log2FC| ≥ 1 and a statistical significance of *P* < 0.05.

**Fig. 8:**
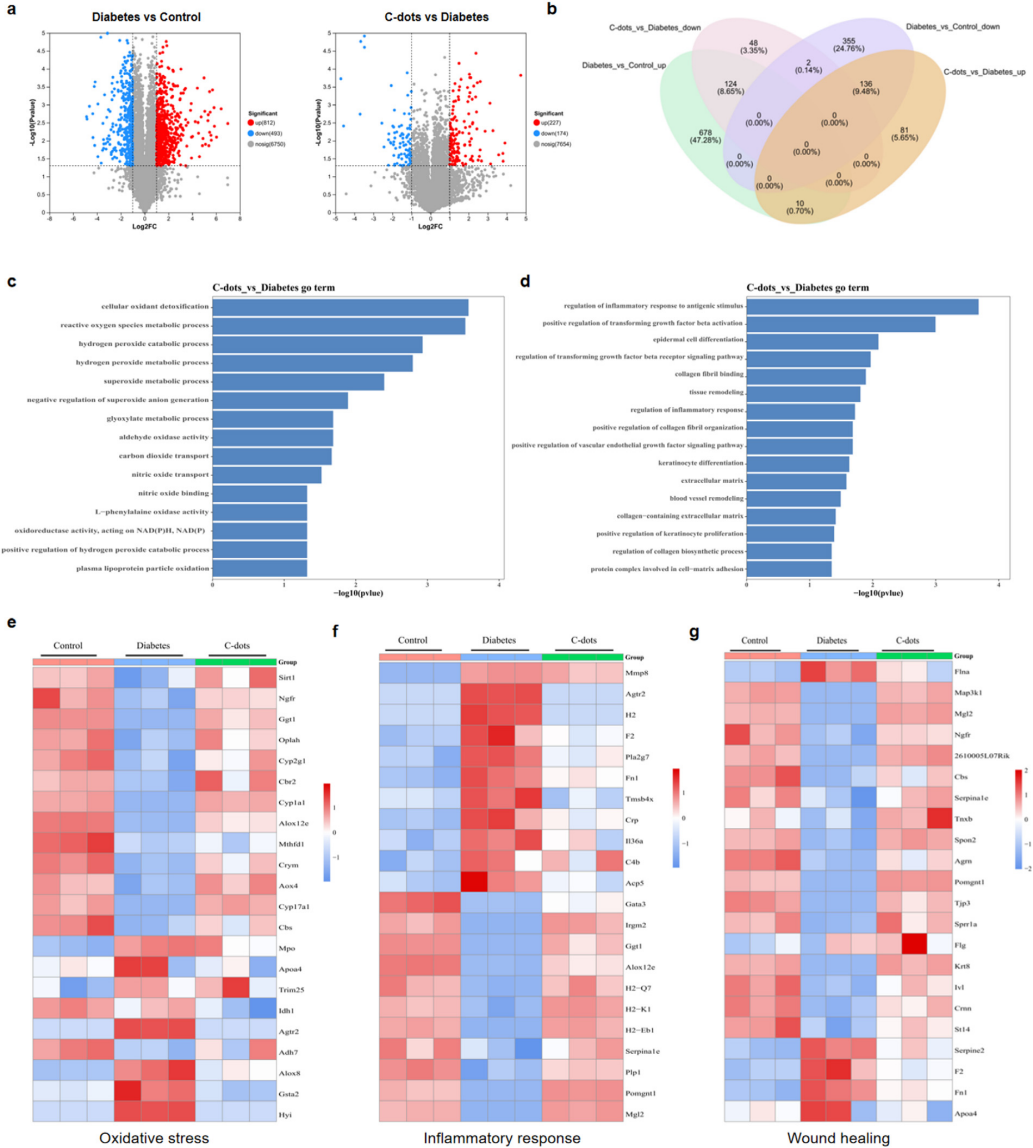
**Proteomic analysis of wound tissues with C-dots treatment. a** Volcano plots illustrating the DEPs between the Diabetes group and the Control group, as well as between the C-dots group and the Diabetes group. **b** Venn diagrams depicting the overlap of DEPs. **c** GO enrichment analysis of biological processes related to oxidative stress. **d** GO enrichment analysis of biological processes associated with wound healing, including inflammation regulation, tissue remodeling, and other relevant processes. **e-g** Heatmaps of proteins associated with oxidative stress (**e**), inflammatory responses (**f**), and the wound healing process (**g**) following C-dots treatment.

The heatmap analysis of differentially expressed proteins (DEPs) after clustering revealed distinct patterns among the Control, Diabetes, and C-dots groups, indicating significant proteomic differences **(Supplementary Fig. 8)**. Comparisons between these groups identified 812 upregulated and 493 downregulated DEPs in the Diabetes group relative to the Control group. When comparing the C-dots group to the Diabetes group, 227 proteins were upregulated and 174 were downregulated **(**Fig. 8**a)**. Venn diagrams indicated that C-dots treatment reversed the expression of 124 upregulated and 136 downregulated DEPs caused by the diabetic state **(**Fig. 8**b).** These results suggest that C-dots improve diabetic wound healing by correcting specific proteomic changes caused by diabetes.

Subsequently, we conducted a functional analysis on these overlapping DEPs using Gene Ontology (GO) analysis. The analysis indicated enrichment in various biological processes related to oxidative stress, such as the reactive oxygen species metabolic process, negative regulation of superoxide anion generation, and oxidoreductase activity regulation. This suggests a role for C-dots in maintaining cellular redox homeostasis **(**Fig. 8**c)**. Additionally, pathways related to the regulation of inflammatory responses, tissue remodeling, blood vessel remodeling, and the extracellular matrix were significantly enriched. These biological processes are closely associated with the wound healing process **(**Fig. 8**d)**.

To graphically represent our findings, heatmap analyses were conducted on DEPs associated with oxidative stress. The C-dots group exhibited a notable reversal of the expression patterns triggered by diabetes, indicating a potential therapeutic effect in countering the proteomic disruptions linked to oxidative stress. C-dots treatment markedly upregulated the expression of certain antioxidant proteins, including Sirt1, Ggt1, Oplah, and Cbr2, which were suppressed in the diabetic state **(**Fig. 8**e)**. In parallel, the heatmap for inflammation demonstrated a significant reduction in the expression of proteins that were often elevated in the inflammatory activation state, such as Il36a, Crp, and Pla2g7 **(**Fig. 8**f)**. These observations further substantiate the anti-inflammatory and antioxidant capabilities of C-dots.

Finally, heatmap analyses were conducted on proteins involved in the proliferative and remodeling phases of wound healing. Specifically, tissue remodeling-related proteins, including Mgl2 and Cbs, and extracellular matrix-related proteins, such as Serpina1e, Spon2, Agrn, and Pomgnt1, were upregulated with the application of C-dots. This upregulation also extended to proteins involved in endothelial growth factor signaling, like Tnxb and Tjp3, as well as proteins involved in fibroblast migration, such as 2610005L07Rik and Ngfr. Additionally, markers of keratinization, including Sprr1a, Krt8, and Ivl, were downregulated in the diabetes group but were upregulated following C-dots treatment **(**Fig. 8**g)**.

In conclusion, our comprehensive proteomic analysis and subsequent functional studies provide compelling evidence of the therapeutic efficacy of C-dots in diabetic wound healing. The ability of C-dots to modulate the expression of key proteins involved in oxidative stress, inflammatory response, and wound healing underscores their potential for improving wound outcomes in diabetic conditions.

## Discussion

In this study, we demonstrated that C-dots notably decrease intracellular ROS within keratinocytes, vascular endothelial cells, and fibroblasts. They serve to shield cells from damage caused by oxidative stress and enhance the migration of fibroblasts. Moreover, the topical administration of C-dots considerably speeds up the healing process of diabetic wounds in a diabetic mouse model. C-dots efficiently neutralize excessive ROS *in vivo*, alleviating oxidative stress in the wound environment. Furthermore, they encourage the transition of macrophages from an M1 to an M2 phenotype, and stimulate neoangiogenesis and collagen formation. These actions collectively facilitate the progression to the proliferative phase of wound healing.

Previous reports have shown that nanomaterials for diabetic wound healing can mitigate oxidative stress, modulate inflammation and promote collagen deposition to speed up the healing process [12], [54]. Our study stands out due to the utilization of C-dots, which are characterized by their ease of synthesis and modification, tunable surface chemistry, high stability, and exceptional biocompatibility. Unlike some other nanomaterials that may have catalase or peroxidase activities, C-dots have been found to possess significant SOD-like activity. In addition to the traditional DHE probe for detecting ROS clearance, we also measured the levels of oxidative stress markers, 8-OHdG and iNOS. 10.13039/100014337Furthermore, we examined macrophage phenotypes to understand how C-dots influence the transition from inflammation to proliferation, a distinct approach from other materials. To elucidate the specific role of C-dots in regulating wound healing, we performed a proteomic analysis, which revealed that C-dots could modulate crucial pathways and proteins associated with oxidative stress, inflammatory responses, and wound healing. These findings robustly support the concept that C-dots exert a suppressive influence on oxidative stress and inflammation while promoting tissue remodeling activities that are essential during the wound repair process.

In this study, a series of experiments was designed to demonstrate C-dots’ efficacy in scavenging ROS. ESR spectroscopy was employed to track the O_2_^•−^ signal in a controlled reaction, with a diminished signal in the presence of C-dots indicating successful detoxification of O_2_^•−^. The WST-1 SOD assay kit was used to detect that C-dots possess high SOD-like activity, capable of scavenging O_2_^•−^. *In vitro* assays using DCFH-DA and MitoSOX demonstrated a reduction in fluorescence intensity in cells treated with C-dots, confirming their ability to scavenge intracellular and mitochondrial ROS. For *in vivo* assessment, an animal model was utilized to evaluate the antioxidant properties of C-dots. After harvesting the tissue and preparing fresh tissue slices, we used the DHE probe for detecting O_2_^•−^ in the tissues. The red fluorescence intensity serves as an indicator of O_2_^•−^ levels. Moreover, proteomic analysis underscored C-dots’ role in regulating proteins associated with oxidative stress pathways, further highlighting their potential to combat oxidative stress.

Wound healing is an extremely complex process involving the activity of various types of cells. Post-injury, keratinocytes migrate to cover wounds, aiding re-epithelialization and releasing growth factors for repair. Endothelial cells stimulate angiogenesis, creating new capillaries for oxygen and nutrient delivery. Fibroblasts produce collagen and matrix, differentiating into myofibroblasts to support wound contraction and tissue regeneration [55]. In our study, we discovered that C-dots exhibited no significant cytotoxicity to these three types of cells, significantly improved cell survival under oxidative stress, and sped up fibroblast migration. These benefits likely stem from C-dots’ antioxidant capabilities, which reduce ROS cytotoxicity and foster a favorable environment for cell repair and regeneration.

In diabetes, the shift of macrophages from the proinflammatory M1 state to the anti-inflammatory M2 state is hampered, leading to an excess of M1 macrophages in wounds and a continuous cycle of inflammation. This cycle inhibits angiogenesis, which is crucial for delivering nutrients and supporting granulation tissue growth during healing. However, C-dots have been shown to promote angiogenesis, as evidenced by an increased number of mature blood vessels in the C-dots treatment group. Additionally, C-dots modulate macrophage phenotypic shifts from M1 to M2, potentially aiding the transition of diabetic wounds from inflammation to proliferation. These findings explain the superior healing outcomes and shorter healing times observed in the C-dots group [52].

Earlier research has evaluated the toxicity of C-dots by administering therapeutic doses via intraperitoneal injection in healthy mice over a 30-day period, revealing no adverse effects on blood or organ health [31]. In the current study, we further examined the safety of C-dots through topical application on mouse skin wounds for the same duration. Similarly, no significant adverse effects were observed in major organs or blood tests. While these findings demonstrate the initial safety of C-dots, long-term exposure to C-dots may have unforeseen impacts on cellular and tissue integrity, which could complicate their clinical translation. Therefore, continued investigation is necessary to ensure their safe and effective application by evaluating the chronic toxicity of C-dots across various cell types and biological models, as well as elucidating their biodistribution, metabolic pathways, and clearance mechanisms. Besides, in light of the current absence of precise targeting features in C-dots, there exists a potential for unintended interactions with non-target cells or tissues. Future studies should explore strategies to enhance the targeting ability of C-dots, such as through surface modifications, to mitigate the risks associated with off-target effects [56], [57].

Although our study has advanced the understanding of C-dots’ potential in diabetic wound healing, especially in terms of oxidative stress modulation, it has not thoroughly investigated the significant role of cytokines. Recognizing their essential part in managing the wound healing process, we are committed to encompass a detailed examination of cytokine profiles and cellular differentiation markers in forthcoming studies. Moreover, the increased susceptibility of diabetic ulcers to bacterial infections, a critical area not fully addressed in our current research, will be a focus of future work. We plan to explore the combination of C-dots with proven antimicrobial agents, such as silver nanoparticles, to leverage their potential synergistic antibacterial effects. These efforts aim to enhance the therapeutic capabilities of C-dots, rendering them a more potent tool for the prevention and management of bacterial infections in diabetic wounds.

## Conclusions

In conclusion, we demonstrated that C-dots could exhibit significant therapeutic potential for diabetic wound healing. C-dots effectively scavenged intracellular ROS and mtROS in HaCaT cells, HUVECs, and fibroblasts. In a diabetic mouse model, C-dots accelerated wound healing by promoting re-epithelialization and collagen deposition, which improved the overall histological appearance of wound tissues. C-dots significantly mitigated oxidative stress in wound tissue, as evidenced by reduced DHE staining and quantitative analysis of 8-OHdG and iNOS levels. They also reduced the expression of pro-inflammatory cytokines TNF-α and IL-6, promoting the polarization of macrophages from the pro-inflammatory M1 phenotype to the anti-inflammatory M2 phenotype. Furthermore, C-dots enhanced angiogenesis in diabetic wounds, as demonstrated by increased expression of CD31 and α-SMA, markers of mature blood vessels. While these findings are promising, they also highlight the challenges in translating preclinical success into clinical practice, including ensuring the safety and efficacy of C-dots in humans, addressing scalability for large-scale production, and obtaining regulatory approval. Addressing these challenges is crucial for the clinical translation of C-dots and unlocking their full therapeutic potential in diabetic wound treatment, with future work focusing on optimizing C-dots properties and advancing towards clinical trials.

## Author contributions

Cui Liu, Zhu Yan, and Yumin Xia designed the experiments. Zhu Yan, Yufei Zhang, and Qin Chen performed the experiments. Zhu Yan analyzed the data and prepared figures. Zhu Yan and Cui Liu wrote the original manuscript. Jing Li, Xiaoying Ning, Fan Bai, and Yaqi Wang provided technical support. Xiaoming Liu, Yale Liu, Mingzhen Zhang, Cui Liu, and Yumin Xia contributed to the review and revision of this manuscript.

## Declaration of competing interest

The authors declare that they have no known competing financial interests or personal relationships that could have appeared to influence the work reported in this paper.
