# Supplementary material for: Carbon dot superoxide dismutase nanozyme enhances reactive oxygen species scavenging in diabetic skin wound repair
Source: J Adv Res. 2025 Mar 26;79:691–706. doi: 10.1016/j.jare.2025.03.049 (PMC12766211; doi:10.1016/j.jare.2025.03.049)
Supplement: Supplementary Data 1 [file mmc1.docx]

**Supplementary Information**

**Carbon dot superoxide dismutase nanozyme enhances reactive oxygen species scavenging in diabetic skin wound repair**

Zhu Yan^a^, Yufei Zhang^a^, Qin Chen^a^, Jing Li^b^, Xiaoying Ning^a^, Fan Bai^a^, Yaqi Wang^c^, Xiaoming Liu^d^, Yale Liu^a^, Mingzhen Zhang^e^, Cui Liu^b,*^, and Yumin Xia^a,*^

*^a^ Department of Dermatology, The Second Affiliated Hospital of Xi’an Jiaotong University, Xi’an 710004, China.*

*^b^ Chongqing Key Laboratory of Natural Product Synthesis and Drug Research, Innovative Drug Research Center, School of Pharmaceutical Sciences, Chongqing University, Chongqing 400044, China.*

*^c^ Department of Dermatology, The Second Affiliated Hospital of Zhejiang University School of Medicine, Hangzhou 310009, China.*

*^d^ Department of Dermatology, Southern University of Science and Technology Hospital, Shenzhen 518055, China.*

*^e^ School of Basic Medical Sciences, Xi'an Jiaotong University, Xi'an 710061, China.*

^*^Corresponding authors: liucui@cqu.edu.cn (C. Liu), xiayumin1202@163.com (Y. Xia)

**Supplementary Methods**

***Instrumentation***

Transmission electron microscopy (TEM) images were captured using FEI Tecnai G2 F30 microscope (USA). Electron spin resonance (ESR) signals were detected using Bruker A300-9.5/12 spectrometer (Germany). X-ray photoelectron spectroscopy (XPS) was conducted with Escalab 250Xi instrument from Thermo Fisher Scientific (USA). Flow cytometric analyses were performed on Becton Dickinson FACS Calibur^TM^ instrument (USA). Absorbance readings from microplate assays were obtained by Tecan Spark 20 M reader (Switzerland).

***Measurement of ESR***

For the detection of O_2_^•−^ scavenging activity, graphite carbon nitride was dispersed in methanol to a concentration of 0.5 mg/mL. We took 50 µL of this solution, added 10 µL of pure DMPO and 40 µL of methanol, mixed well, and then exposed it to 300W xenon lamp light for 5 minutes to collect data as the control group data. For the test of C-dots, we replaced the 40 µL of methanol with 40 µL of the methanol dispersion of C-dots.

For the detection of •OH scavenging activity, a solution of FeSO4 was prepared at a concentration of 5 mg/mL. 100 µL of this solution was taken and 10 µL of pure DMPO and 80 µL of deionized water were added. Upon the addition of 10 µL of 30% H_2_O_2_ solution, the mixture was thoroughly mixed and then permitted to react for a period of 5 minutes. A sample was taken for testing, and this data was used as the control group data. For the test of C-dots, 80 µL of deionized water was replaced with 80 µL of the C-dots solution.

For assessing •NO scavenging activity, a PBS solution of SNAP at a concentration of 10 mM , and a PBS solution of Carboxy-PTIO at a concentration of 2 mM were prepared. 20 µL of the SNAP solution were mixed with 20 µL of the PTIO and 20 µL of PBS, and the mixture was given a reaction time of 10 minutes. Data were collected from this reaction and used as the control group data. For the test of C-dots, 20 µL PBS was replaced with 20 µL C-dots solution, and data were collected accordingly.

***In vitro cytotoxicity assay for C-dots***

To evaluate the cytotoxicity of C-dots, HaCaT cells, fibroblasts, and HUVECs were seeded in 96-well plates and incubated for 24 hours. Subsequently, the cells were exposed to varying concentrations of C-dots for 24 and 48 hours. Cytotoxicity was evaluated using a CCK-8 assay kit (Beyotime, Shanghai, China), following the protocol provided by the supplier. The optical density at 450 nm was assessed using a plate reader to evaluate the influence of C-dots on the cells’ survival rate.

***Hemocompatibility assay***

Following the reported procedures, we evaluated the hemocompatibility of C-dots using a hemolysis test [[1-3](#_ENREF_1)]. Mouse blood was collected and diluted with PBS from 2 mL to 4 mL. Following centrifugation at 3500 rpm for 5 minutes, red blood cells (RBCs) were collected, isolated, and rinsed with PBS to eliminate the red hue from the supernatant. The purified RBCs were then resuspended in 20 mL of PBS. Equal volumes (800 µL) of C-dots at varying concentrations were prepared in PBS and mixed with 200 µL of the diluted RBC suspension. PBS served as a negative control, and ultrapure water functioned as a positive control. In addition to the experimental group (C-dots + RBC), the C-dots only group was included to exclude their own absorbance. After incubating for 4 hours at 37°C, the samples were spun at 3500 rpm for 5 minutes. The resulting supernatant was then pipetted into a 96-well plate, and the absorbance at 540 nm was measured using a microplate reader. Hemolytic quantification was performed as follows: Hemolysis (%) = [(As– An)/(Ap–An)] × 100%, where As represented the difference between the absorbance of the supernatant from the C-dots + RBC group and that of the C-dots only group at the same concentration, Ap denoted the absorbance of the positive control, and An signified the absorbance of the negative control.

***Proteomics analysis***

Mouse wound tissue samples were homogenized to process the total protein extraction, which was then quantified using the BCA assay. After undergoing pancreatic enzymatic hydrolysis, the resulting peptide concentrations were determined with a NANO DROP ONE spectrophotometer from Thermo Fisher Scientific (Waltham, MA, USA). For Data-Independent Acquisition (DIA) mass spectrometry analysis, peptide samples were separated on a VanquishNeo chromatograph from Thermo. The mass spectrometry analysis was conducted on an Astral mass spectrometer from Thermo. The DIA data files were subsequently processed using Spectronaut™ software (Biognosys AG, Version 14). Bioinformatics tools and databases accessible on the majorbio cloud platform (cloud.majorbio.com) were utilized to perform functional annotation and pathway analysis of the proteins, shedding light on the biological significance of changes in protein expression. Finally, statistical analysis of the data provided further interpretation of these changes.

**Supplementary figures**

**
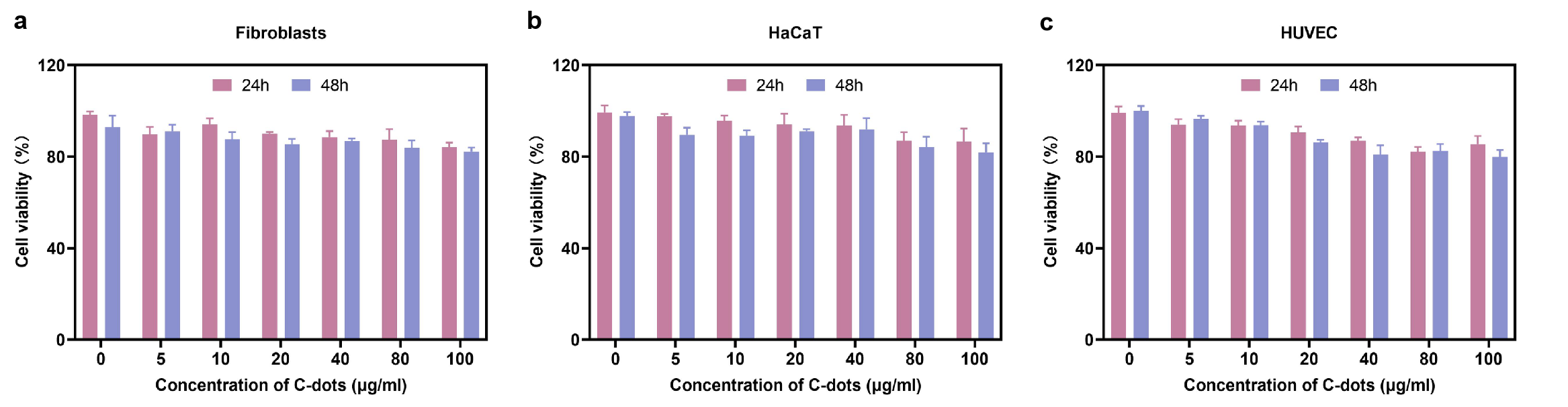
**

**Supplementary Fig. 1. *In vitro* cytotoxicity assay for C-dots. a-c** Cell viabilities of fibroblasts (**a**), HaCaT cells (**b**), and HUVECs (**c**) following 24 and 48 hours of co-incubation with C-dots at varying concentrations. Data are presented as means ± SEM (n = 3).

**
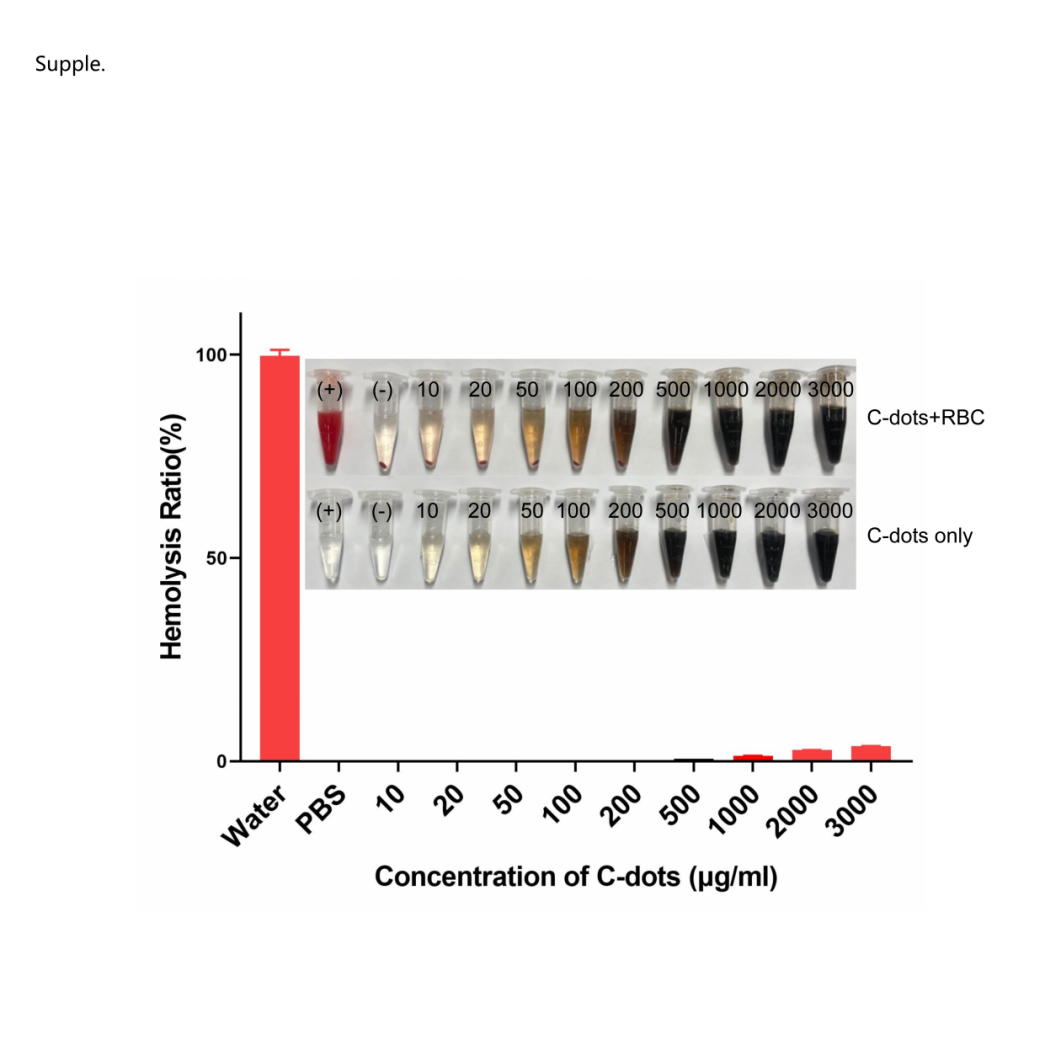
**

**Supplementary Fig. 2. Hemolysis ratio of erythrocytes induced by C-dots at varying concentrations**. The C-dots + RBC group was the experimental group, showing water (+), PBS (-), and different concentrations of C-dots with red blood cell suspension. The C-dots only group served as the material control group, containing only C-dots at the same concentrations. Data are presented as means ± SEM (n = 3).


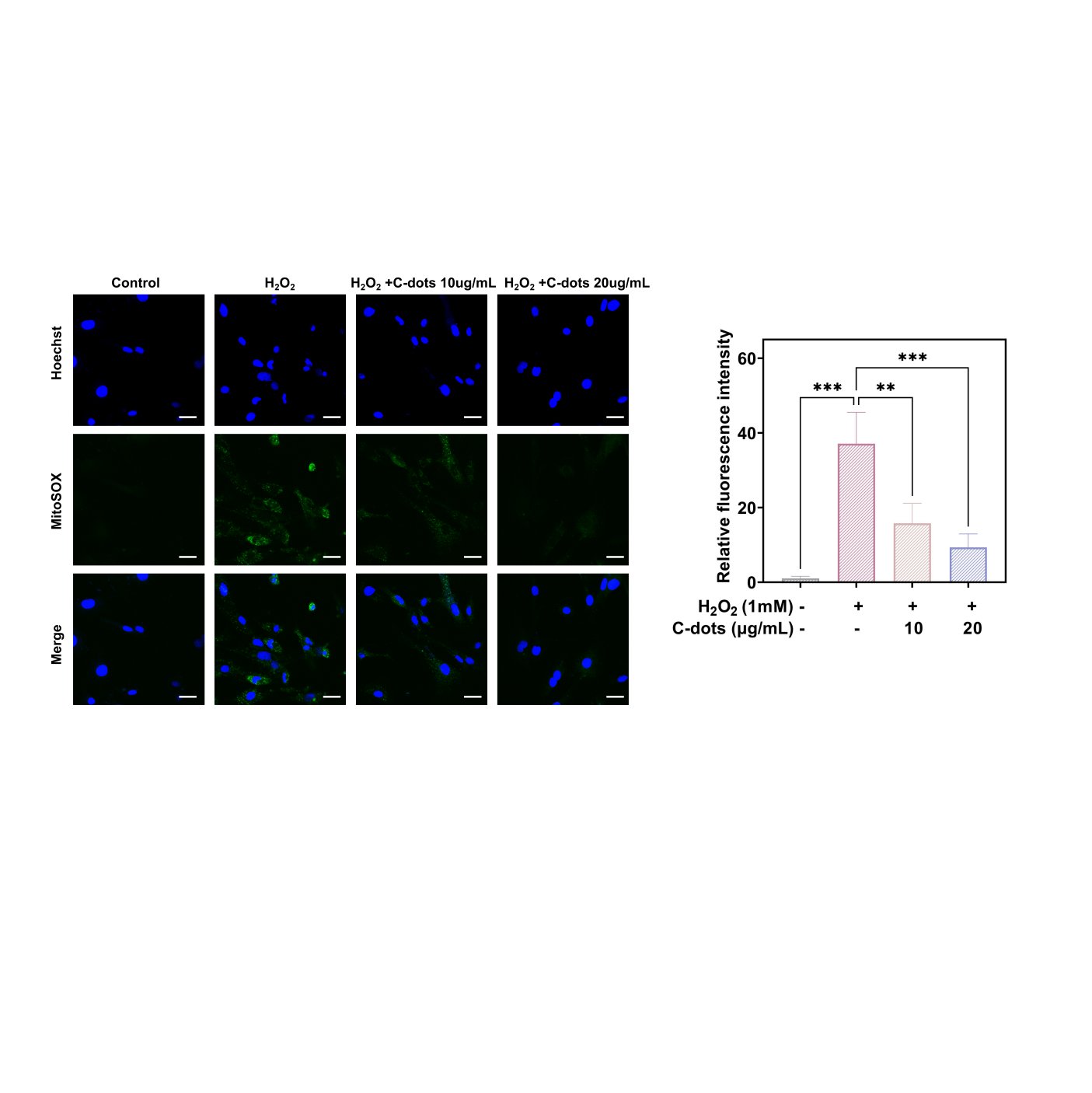


**Supplementary Fig. 3. Scavenging effect of C-dots on mitochondrial ROS in fibroblasts.** C-dots significantly attenuated H₂O₂-induced mitochondrial ROS accumulation in fibroblasts, as quantified by MitoSOX Green staining. Data are presented as means ± SEM (n = 3; * *P* < 0.05, ** *P* < 0.01, *** *P* < 0.001, **** *P* < 0.0001). Scale bar = 25 µm.





**Supplementary Fig. 4. Comparative photographs of C-dots@H_2_O, Lotion, and C-dots@Lotion.** Visual images of different formulations: C-dots suspended in water (C-dots@H_2_O), a standard lotion, and a combination of C-dots with lotion (C-dots@Lotion).





**Supplementary Fig. 5. Body weight measurements of mice.** Monitoring the body weight measurements of mice in different groups over a period of time. Data are presented as means ± SEM (n = 5).


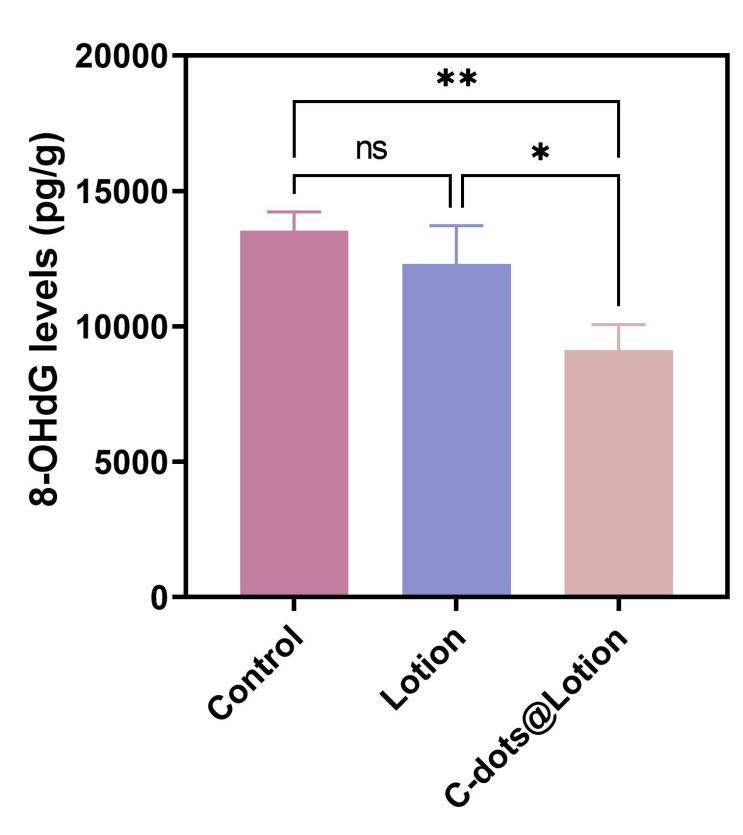


**Supplementary Fig. 6. Quantification of 8-OHdG levels in wound tissues across all groups using ELISA.** The C-dots@Lotion group showed significantly lower 8-OHdG levels compared to both the control and lotion groups, demonstrating C-dots’ antioxidant capacity. Data are presented as means ± SEM (n = 5; * *P* < 0.05, ** *P* < 0.01, *** *P* < 0.001, **** *P* < 0.0001).


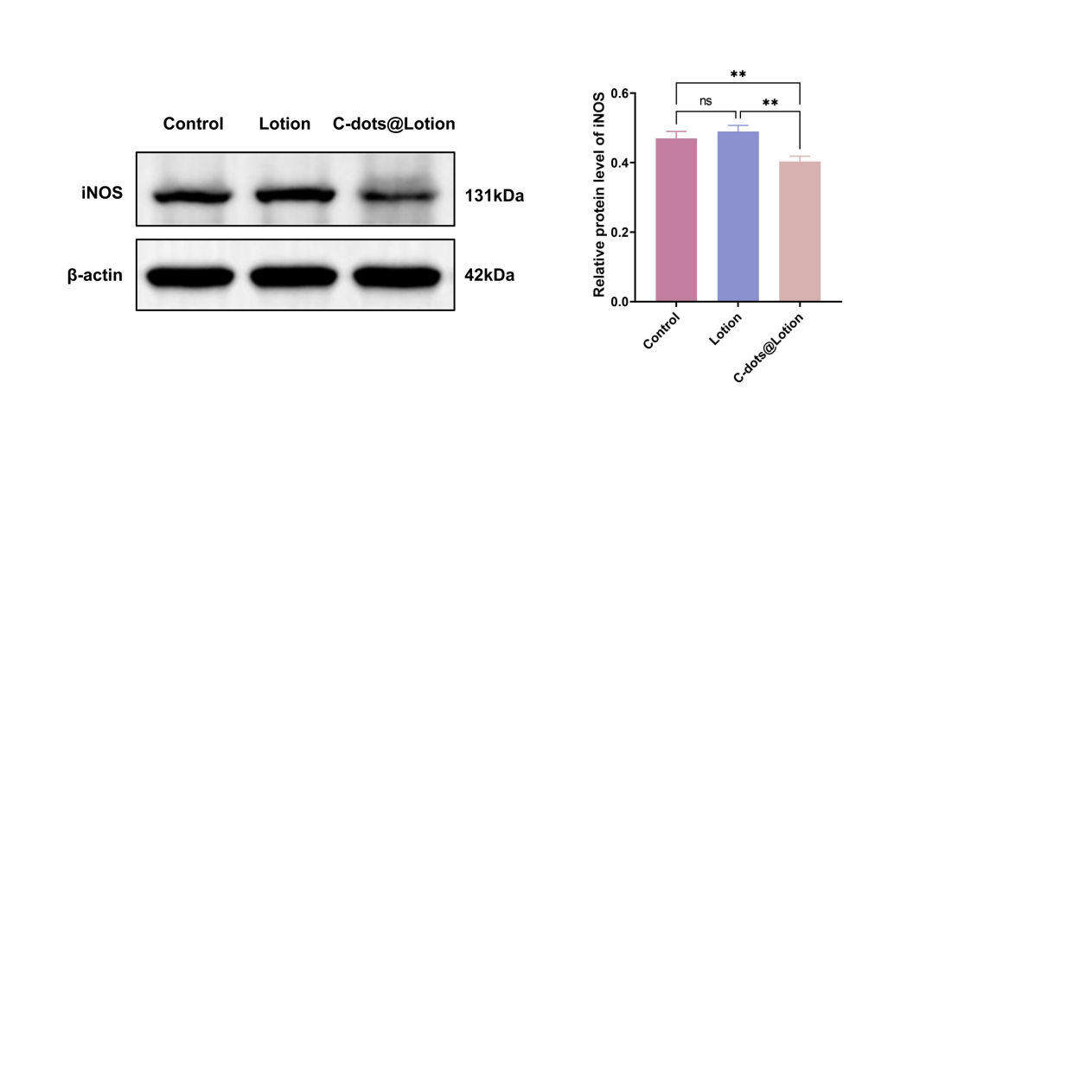


**Supplementary Fig. 7.** **Western blotting analysis of iNOS protein expression in wound tissue from each group.** Quantitative analysis showed C-dots significantly reduced iNOS levels in wound tissue. Data are presented as means ± SEM (n = 3; * *P* < 0.05, ** *P* < 0.01, *** *P* < 0.001, **** *P* < 0.0001).


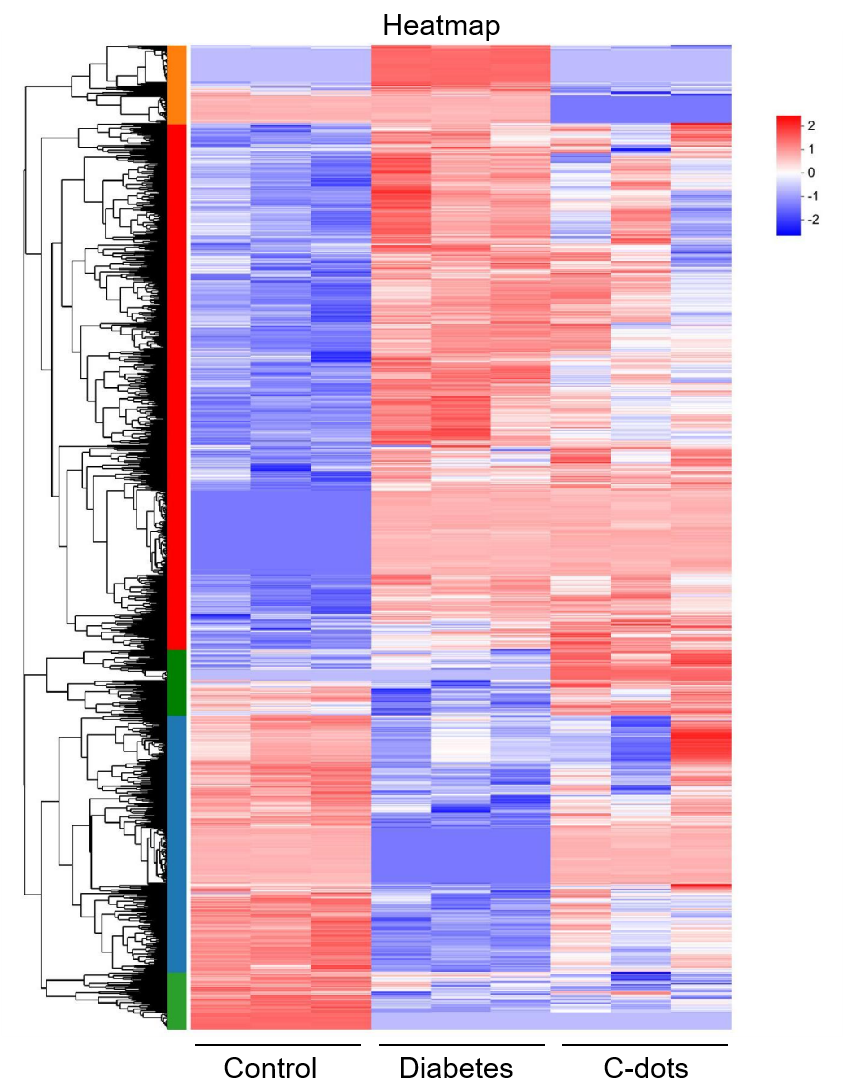


**Supplementary Fig. 8.** **Heatmap of proteomic changes with C-dots treatment.** The heatmap displays the expression levels of differentially expressed proteins (DEPs) across three mouse groups: non-diabetic healthy controls (Control), STZ-induced diabetic mice (Diabetes), and C-dots@Lotion treated diabetic mice (C-dots), which underscores the therapeutic effect of C-dots in modulating the proteomic response in diabetic wound healing.

**References**

1. Han X, Saengow C, Ju L, Ren W, Ewoldt RH, Irudayaraj J. Exosome-coated oxygen nanobubble-laden hydrogel augments intracellular delivery of exosomes for enhanced wound healing. Nature communications. 2024; 15: 3435.

2. Li S, Li X, Xu Y, Fan C, Li ZA, Zheng L, et al. Collagen fibril-like injectable hydrogels from self-assembled nanoparticles for promoting wound healing. Bioactive Materials. 2024; 32: 149-63.

3. Sun X, Wang P, Tang L, Li N, Lou YR, Zhang Y, et al. Multifunctional hydrogel containing oxygen vacancy‐rich wox for synergistic photocatalytic O2 production and photothermal therapy promoting bacteria‐infected diabetic wound healing. Advanced Functional Materials. 2024; 34: 2411117.
